# Inhibition of Japanese encephalitis virus infection by the host zinc-finger antiviral protein

**DOI:** 10.1371/journal.ppat.1007166

**Published:** 2018-07-17

**Authors:** Hsin-Ping Chiu, Han Chiu, Chao-Fu Yang, Yi-Ling Lee, Feng-Lan Chiu, Hung-Chih Kuo, Ren-Jye Lin, Yi-Ling Lin

**Affiliations:** 1 Graduate Institute of Microbiology, National Taiwan University, Taipei, Taiwan; 2 Institute of Biomedical Sciences, Academia Sinica, Taipei, Taiwan; 3 Institute of Cellular and Organismic Biology, Academia Sinica, Taipei, Taiwan; 4 National Mosquito-Borne Diseases Control Research Center, National Health Research Institute, Taipei, Taiwan; 5 Genomics Research Center, Academia Sinica, Taipei, Taiwan; University of Texas Southwestern Medical Center at Dallas, UNITED STATES

## Abstract

CCCH-type zinc-finger antiviral protein (ZAP) is a host factor that restricts the infection of many viruses mainly through RNA degradation, translation inhibition and innate immune responses. So far, only one flavivirus, yellow fever virus, has been reported to be ZAP-resistant. Here, we investigated the antiviral potential of human ZAP (isoform ZAP-L and ZAP-S) against three flaviviruses, Japanese encephalitis virus (JEV), dengue virus (DENV) and Zika virus (ZIKV). Infection of JEV but not DENV or ZIKV was blocked by ZAP overexpression, and depletion of endogenous ZAP enhanced JEV replication. ZAP hampered JEV translation and targeted viral RNA for 3′-5′ RNA exosome-mediated degradation. The zinc-finger motifs of ZAP were essential for RNA targeting and anti-JEV activity. JEV 3′-UTR, especially in the region with dumbbell structures and high content of CG dinucleotide, was mapped to bind ZAP and confer sensitivity to ZAP. In summary, we identified JEV as the first ZAP-sensitive flavivirus. ZAP may act as an intrinsic antiviral factor through specific RNA binding to fight against JEV infection.

## Author summary

In addition to innate and adaptive immunities, many cellular proteins also exert antiviral activity against viral invasion. Human zinc-finger antiviral protein (ZAP) is a cellular restriction factor against many viruses but its role with regard to the flavivirus family is largely unknown. We tested the antiviral potential of ZAP against three flaviviruses and found that Japanese encephalitis virus (JEV) was ZAP-sensitive, while dengue virus and Zika virus were ZAP-resistant. ZAP specifically targets JEV viral RNA and induces translation repression and RNA degradation. Our findings highlight the ZAP-mediated anti-JEV mechanisms and extend the antiviral spectrum of ZAP to include a member of the Flavivirus genus.

## Introduction

Zinc-finger CCCH-type containing, antiviral 1 (ZC3HAV1) also known as zinc-finger antiviral protein (ZAP) was first discovered in rats as a host antiviral protein against Moloney murine leukemia virus [1]. Later, multiple RNA and DNA viruses, like retroviruses, filoviruses, alphaviruses, and hepatitis B virus were shown to display sensitivity to ZAP [2–6]. However, ZAP does not induce a universal antiviral state, since viruses such as herpes simplex virus 1 (HSV-1), vesicular stomatitis virus and yellow fever virus (YFV) are resistant to ZAP [4]. Furthermore, viruses within the same family may exhibit different sensitivity to ZAP. For example, in the family *Picornaviridae*, ZAP inhibited coxsackievirus B3 but not poliovirus infection [4, 7].

In humans, ZAP contains two major isoforms (ZAP-L and ZAP-S), which differ in the C-terminal region through alternative splicing [8]. In the N-terminus of ZAP, there are four CCCH-type zinc-finger motifs, which are required for RNA binding and antiviral property [9]. ZAP exhibits antiviral activity generally via posttranscriptional RNA regulation, such as mRNA decay and translation inhibition. ZAP recruits the RNA processing exosome complex and poly(A)-specific ribonuclease (PARN) to degrade the target RNA from the 3′-end [6, 10]. ZAP also interacts with p72 RNA helicase to recruit decapping enzymes DCP1/DCP2 and exoribonuclease XRN1 to degrade the target RNA from the 5′-end [6, 11]. Besides targeting RNA accumulation, ZAP can block translation of incoming viral RNA of Sindbis virus (SINV) [4] probably by interrupting the interaction between translational initiation factors eIF4A and eIF4G [12]. Moreover, ZAP-S can associate with retinoic acid-inducible gene I (RIG-I), a key sensor to recognize viral RNA, to promote the innate immune response and contribute to its antiviral potential [13].

Flaviviruses include numerous important human pathogens such as YFV, West Nile virus (WNV), Zika virus (ZIKV), dengue virus (DENV) and Japanese encephalitis virus (JEV), causing endemic or pandemic outbreaks in tropical and subtropical areas [14]. Flaviviral virions are enveloped and contain a single-stranded, positive-sense RNA genome with 5′-cap but not 3′-poly(A) tail. Flavivirus RNA encodes a single open reading frame (ORF) flanked by 5′- and 3′-untranslated regions (UTRs), which contain RNA secondary structures required for viral translation and transcription [15, 16]. YFV was resistant to the antiviral activity of rat ZAP [4], but the susceptibility of other flaviviruses to ZAP is not clear. In this study, we determined the antiviral activity of ZAP against JEV, DENV, and ZIKV and found different viral responses to ZAP. We further demonstrated the antiviral mechanism of ZAP against JEV infection.

## Results

### Overexpression of ZAP inhibits JEV infection

**Fig 1:**
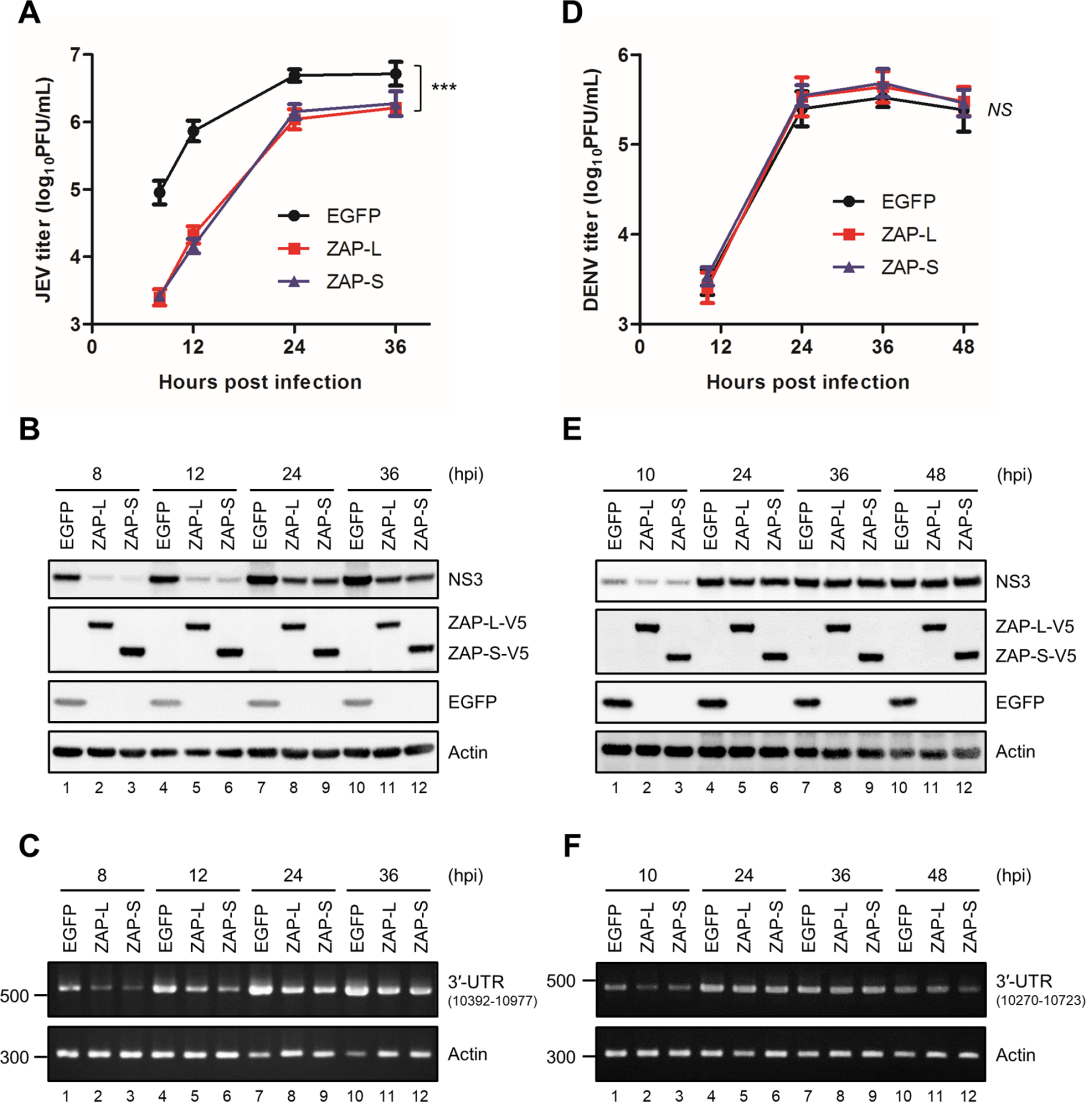
Inhibition of JEV but not DENV infection by human ZAP isoforms. A549 cells transduced with lentiviruses expressing EGFP (A549-EGFP), V5-tagged ZAP-L (A549-ZAP-L) and V5-tagged ZAP-S (A549-ZAP-S) were infected with JEV or DENV (MOI = 5). Culture supernatants, cell lysates and total RNA were harvested at the indicated time points. (A and D) Viral titration determined by plaque assay. Representative data from three independent experiments are shown as mean ± SD (n = 3). Statistical significance was analyzed by two-way ANOVA. *** *P*≤0.001; *NS*, not significant. (B and E) Protein levels of viral NS3, V5-tagged ZAP, EGFP and actin were analyzed by western blot. (C and F) RNA levels of viral genome and actin were performed by RT-PCR. hpi, hours post-infection.

To evaluate the antiviral potential of human ZAP against members of flavivirus, we established A549 cells overexpressing ZAP-L and ZAP-S by lentiviral transduction. Cells with or without ZAP overexpression were infected with JEV, DENV or ZIKV and analyzed for viral replication. As compared with control EGFP cells, JEV infection measured by viral propagation, viral NS3 protein expression, and viral RNA replication were lower in cells ectopically overexpressing ZAP-L and ZAP-S (Fig 1A–1C). The inhibitory effect of ZAP-L/S against JEV infection was also noted in the induced pluripotent stem cells (iPSCs)-derived human neural progenitor cells (hNPCs), human microglia HMC3 cell line, as well as human neuroblastoma BE(2)C and SK-N-SH cell lines (S1 Fig). Nevertheless, no significant antiviral effect of ZAP was observed after high and low multiplicity of infection (MOI) of DENV and ZIKV (Fig 1D–1F, S2 and S3 Figs). Thus, different flaviviruses exhibit different sensitivity to the antiviral activity of ZAP.

Viruses may downregulate the expression of ZAP and/or block its functions to evade the ZAP-mediated antiviral action [17–20]. To understand why DENV was resistant to ZAP, we detected the endogenous protein levels of ZAPs in JEV or DENV-infected cells and no reduction of ZAPs was noted (S4 Fig). The antiviral activity of ZAP against SINV was also not obstructed by DENV infection (S5 Fig), implying that DENV did not antagonize the antiviral activity of ZAP. Other mechanisms, besides affecting ZAP expression and function, need to be considered.

### Downregulation of ZAP enhances JEV replication

**Fig 2:**
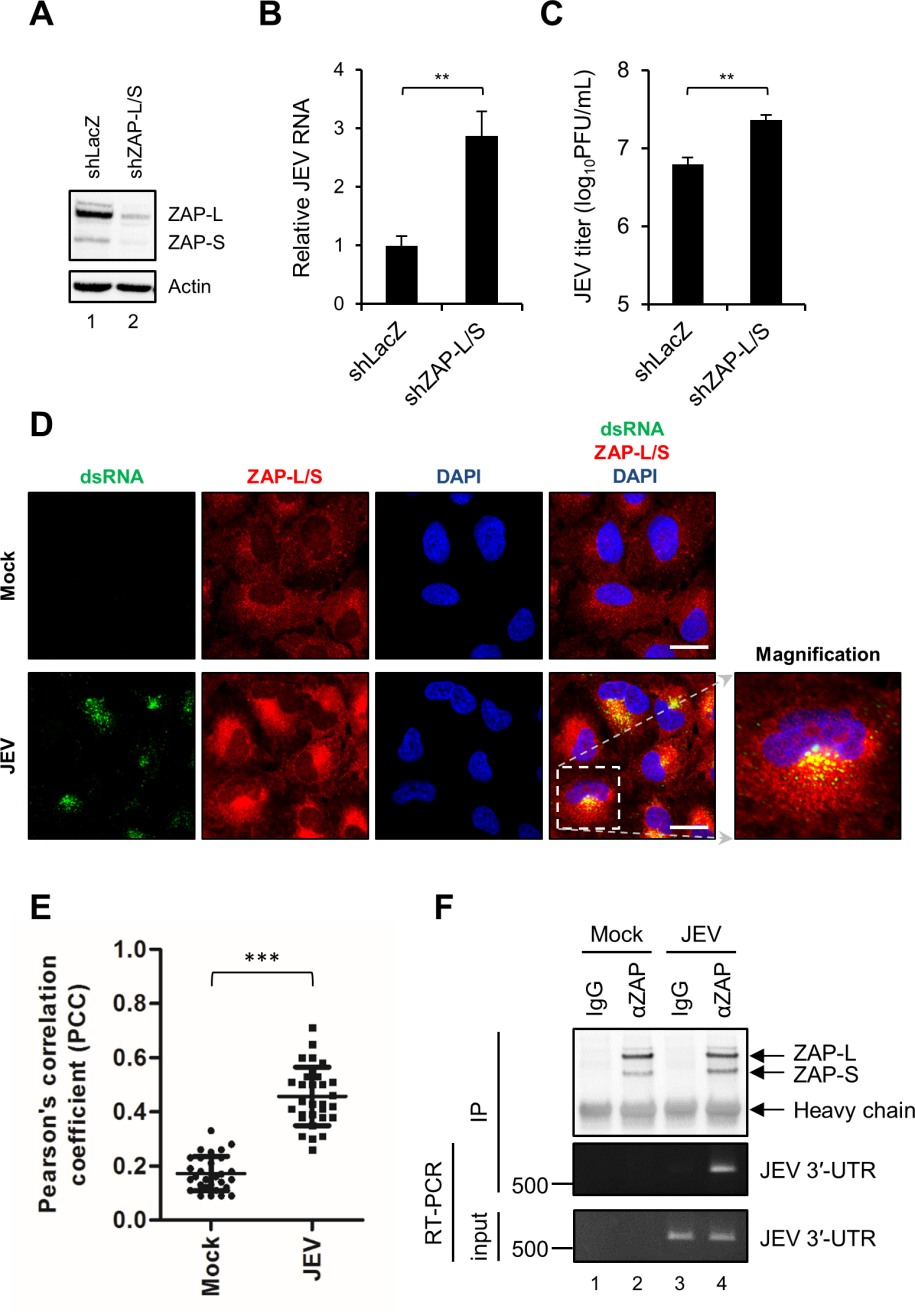
Antiviral potential of endogenous ZAP against JEV infection. (A) Western blot analysis for the expression of ZAP in A549 cells transduced with lentiviruses carrying shRNA targeting LacZ and ZAP-L/S. (B and C) A549-shLacZ and -shZAP-L/S cells were infected with JEV (MOI = 5) for 24 h. Total RNA and culture supernatants were harvested for RT-qPCR (B) and viral titration (C), respectively. Relative JEV RNA level normalized by GAPDH was determined using RT-qPCR. Viral titer was measured using plaque assay. Representative data from two independent experiments shown as mean ± SD (n = 3) were analyzed by two-tailed Student’s *t* test. ** *P*≤0.01. (D) Confocal microscopy of mock and JEV (MOI = 1) infected A549 cells at 16 hpi stained with anti-dsRNA and anti-ZAP antibodies. Cell nuclei were counterstained with DAPI. Scale bar = 20 μm. (E) Co-localization of dsRNA with ZAP was estimated by Pearson’s correlation coefficient (PCC). Mean ± SD was calculated from 30 cells each group and the statistical significance was analyzed by two-tailed Student’s *t* test. *** *P*≤0.001. (F) Cell lysates from mock and JEV (MOI = 1) infected A549 cells after 16 h of infection were incubated with anti-ZC3HAV1 (ZAP) antibody or control IgG. Western blot analysis of the immunoprecipitated ZAP isoforms (upper panel). The ZAP-binding viral RNA pulled down by antibodies was amplified by RT-PCR with JEV 3′-UTR specific primers (middle panel). RT-PCR of input JEV viral RNA (lower panel).

To investigate the antiviral potential of endogenous ZAP, A549-shZAP-L/S cells deprived of ZAP expression were established by transduction with lentiviral vector expressing shRNA targeting both ZAP-L and ZAP-S (Fig 2A). As compared to the control shLacZ cells, shZAP-L/S cells supported significantly higher levels of JEV infection as indicated by a 2.87- and 3.08-fold increase in viral RNA and viral progeny production, respectively (Fig 2B and 2C). Furthermore, knockdown of ZAP in human neuronal BE(2)C cells enhanced JEV replication, supporting the anti-JEV role of endogenous ZAP in neuronal cells (S6 Fig). Since JEV replication occurs at the surface of the endoplasmic reticulum (ER) membrane in the cytoplasm [21], and ZAP predominantly localizes to the cytoplasm [22], we examined whether ZAP co-localized with JEV viral RNA. A549 cells with or without JEV infection were processed for immunofluorescence analysis with anti-dsRNA antibody for viral replication complex [23, 24] and anti-ZAP antibody for endogenous ZAP protein. Co-localization of dsRNA and ZAP was seen at the viral replication sites in JEV-infected cells (Fig 2D and 2E). Furthermore, we applied immunoprecipitation/RT-PCR assay using antibody against endogenous ZAP to evaluate the interaction of ZAP with JEV viral RNA. ZAP-L and ZAP-S precipitated by anti-ZAP antibody also pulled down JEV RNA (Fig 2F), indicating association between ZAP proteins and JEV RNA.

### ZAP interacts with JEV RNA and exerts antiviral activity through its zinc-finger motifs

**Fig 3:**
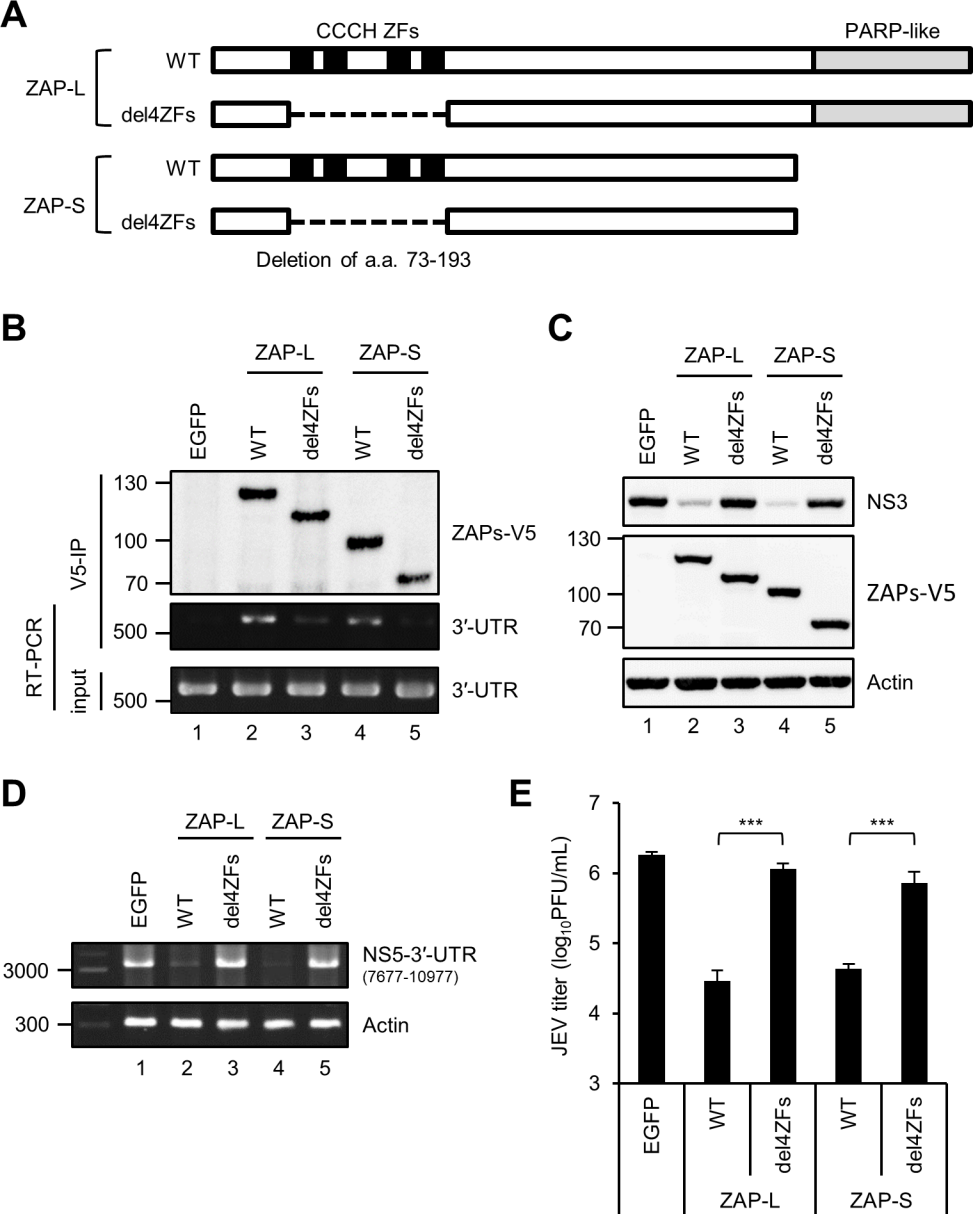
Zinc-finger motifs of ZAP are required for JEV RNA binding and its antiviral activity. (A) Schematic representation of human ZAP isoforms (ZAP-L, 902 a.a. and ZAP-S, 699 a.a.). The four tandem CCCH-type zinc-finger (ZF) motifs within the N-terminus of ZAP are shown as solid black boxes. Deletion of the four ZFs (a.a. 73–193) are indicated with a dashed line. (B) 293T/17 cells infected with JEV (MOI = 5) for 16 h were transfected with plasmids expressing EGFP, WT or ZF-deleted ZAP-L-V5 and ZAP-S-V5 for additional 24 h. The viral RNA bound with V5-tagged proteins was pulled down by anti-V5 agarose affinity gel and amplified by RT-PCR with JEV 3′-UTR specific primers (middle panel). RT-PCR of input viral RNA in JEV-infected cells (lower panel). Western blot analysis of the immunoprecipitated ZAP-L and ZAP-S (WT and del4ZFs) (upper panel). (C-E) The indicated cells were infected with JEV (MOI = 5) for 10 h. Cell lysates, total RNA, and culture supernatants were determined for the indicated proteins by western blot (C), viral RNA by RT-PCR (D), and viral titer by plaque assay (E). Representative data from three independent experiments shown as mean ± SD (n = 3) were analyzed by two-tailed Student’s t test. *** *P*≤0.001.

To understand the anti-JEV mechanism of ZAP, we looked at whether RNA binding is required by generating constructs expressing the zinc-finger (ZF) domains deleted ZAP-L and ZAP-S (ZAP-L/S-del4ZFs) (Fig 3A). We first tested the binding ability of wild type (WT) and ZF-deleted ZAP with JEV viral RNA by immunoprecipitation/RT-PCR assay. The V5-tagged WT ZAP-L and ZAP-S precipitated by anti-V5 antibody also pulled down JEV RNA, which was not seen in ZF-deleted ZAP and EGFP control (Fig 3B). Furthermore, the anti-JEV activity of ZAP was greatly reduced in the ZF-deleted mutants as shown by viral NS3 protein expression, viral RNA replication, and viral progeny production (Fig 3C–3E). Thus, the zinc-finger motifs of ZAP were essential for RNA targeting and antiviral activity against JEV infection. To explore why DENV was resistant to the antiviral action of ZAP, we determined whether ZAP associated with DENV viral RNA. Interestingly, ZAP did not bring down DENV RNA while it readily pulled down the cellular TRAILR4 mRNA (S7 Fig) known to bind with ZAP [25], suggesting that RNA-binding ability may dictate the antiviral potential of ZAP.

### ZAP interferes with viral translation and prevents accumulation of JEV RNA by destabilizing viral RNA

**Fig 4:**
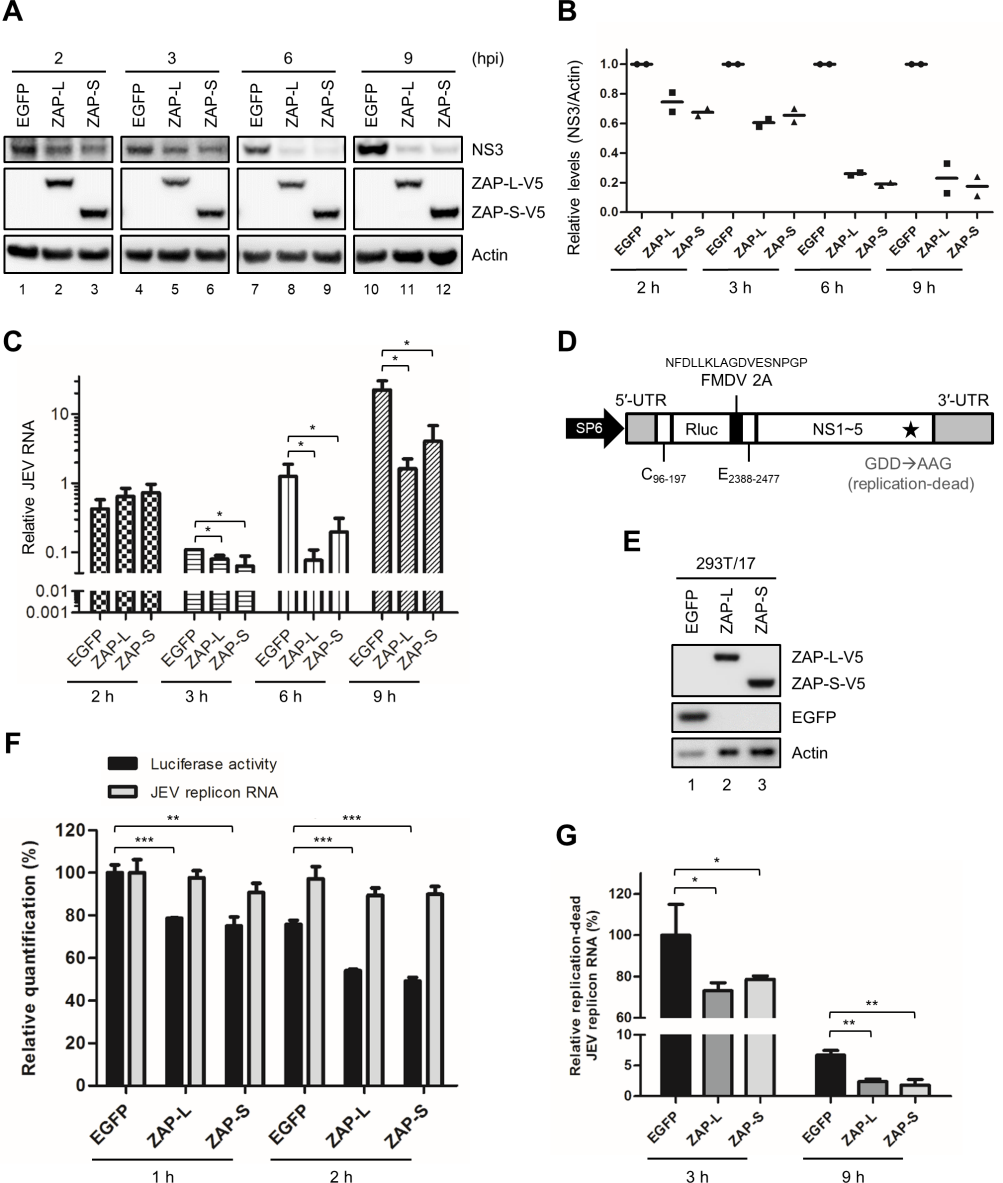
Inhibition of JEV translation and replication by ZAP. (A and C) A549-EGFP, ZAP-L and ZAP-S cells absorbed with JEV (MOI = 10) on ice for 2 h were washed and then incubated at 37°C. (A) At the indicated time points, proteins were harvested for western blot with the indicated antibodies. (B) Relative NS3 protein levels normalized with actin from two independent experiments were quantified by ImageJ software. (C) RNA was collected for viral RNA determination by using RT-qPCR. Relative JEV RNA level was normalized by that of GAPDH. (D) Illustration of SP6 promoter-driven RdRP-dead JEV replicon (E) Western blot of 293T/17 cells expressing EGFP, ZAP-L-V5 and ZAP-S-V5 with the indicated antibodies. (F) 293T/17-EGFP and -ZAP-S cell were cotransfected with 5′-capped RdRP-dead JEV replicon RNA and control firefly luciferase RNA for 1 and 2 h. At the indicated time points, cells were collected and separated into two portions for the measurements of luciferase activity and RNA level, respectively. The relative luciferase activity and RNA level of Renilla luciferase reporter normalized with transfection control firefly luciferase are shown as the percentage to that of EGFP at 1 h post transfection. (G) 5′-capped RdRP-dead JEV replicon RNA and control firefly luciferase RNA cotransfected 293T/17-EGFP and -ZAP-S cells were harvested at 3 and 9 h post-transfection to determine the replicon RNA level. The relative replicon RNA level normalized with that of firefly luciferase is shown. Representative data are shown as mean ± SD from 3 independent experiments and analyzed by two-tailed Student’s t test. * *P*≤0.05; ** *P*≤0.01; *** *P*≤0.001.

After entering the cells, JEV RNA undergoes a first round of translation to produce nonstructural proteins required for viral RNA replication. To assess whether viral translation was affected by ZAP, we detected viral protein expression in ZAP- and EGFP-overexpressing cells at early time points of JEV infection. Lower JEV NS3 protein expression was noted in cells with ZAP overexpression starting from 2 hours post-infection (hpi) (Fig 4A and 4B). We also monitored the viral RNA levels in cells with or without ZAP overexpression by RT-qPCR. No significant difference was found at 2 hpi, but ZAP significantly reduced JEV RNA since 3 hpi (Fig 4C). To further address whether the decrease of viral protein at early time point of JEV infection is through repressing translation and/or reducing viral RNA, we used replication-dead JEV replicon RNA (Fig 4D) to transfect 293T/17 cells with or without ZAP overexpression (Fig 4E). The cell lysates were separated into two portions for measurements of luciferase activity and RNA level. Reduction of luciferase activity was noted in cells with ZAP overexpression since 1 h post-transfection when the RNA level was not yet affected (Fig 4F), suggesting repression of viral translation by ZAP. To clarify whether ZAP influenced JEV viral RNA stability, we also used replication-dead JEV replicon RNA for longer time points. A decrease in replicon RNA was noted at 3 h and 9 h post-transfection in cells with ZAP overexpression as compared to those with EGFP control (Fig 4G), suggesting that ZAP promoted JEV viral RNA decay. Thus, ZAP inhibited JEV translation and impaired viral RNA stability.

### JEV suppression by ZAP is dependent on the 3′-5′ RNA decay pathway

**Fig 5:**
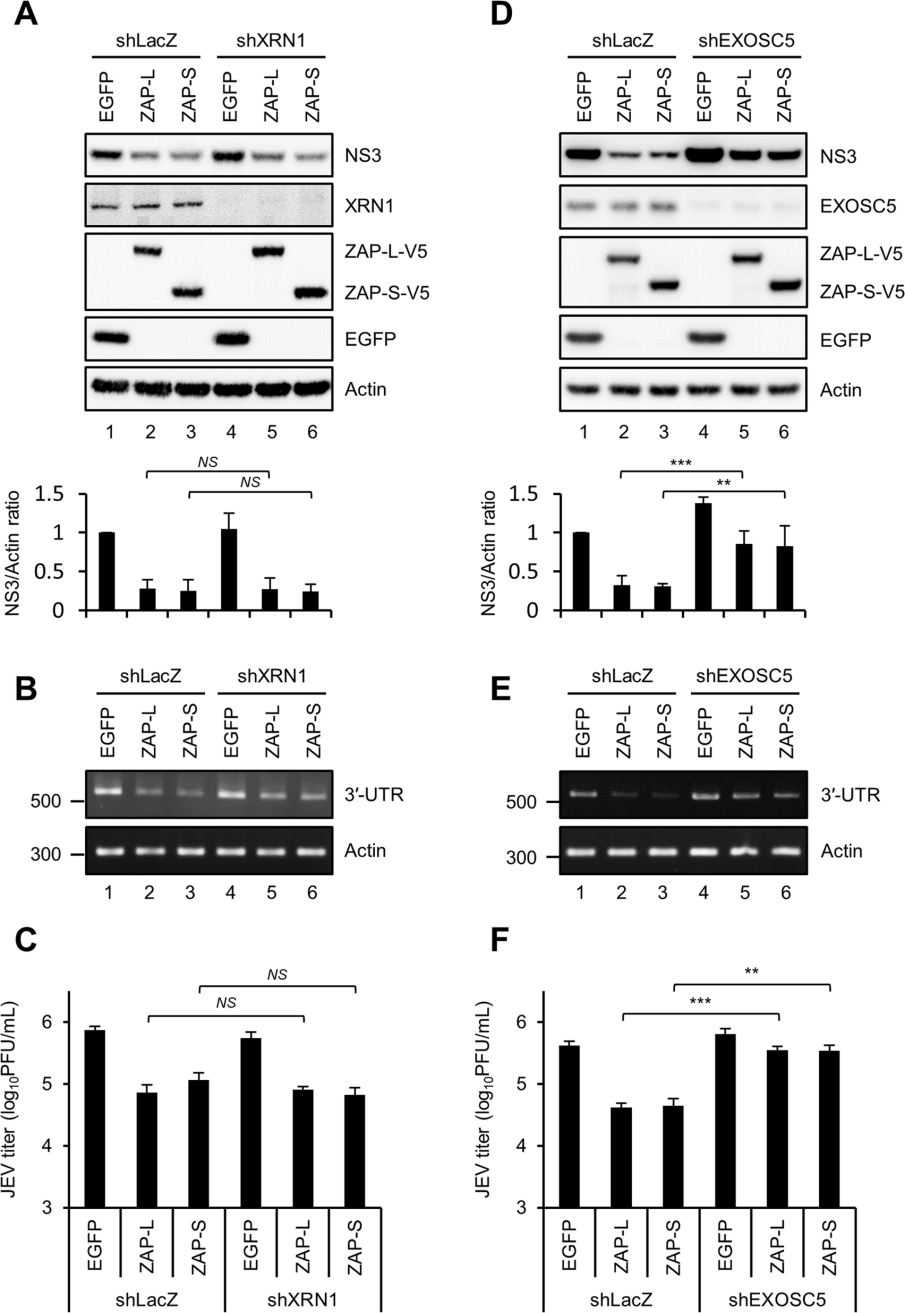
The 3′-5′ RNA exosome-mediated, but not the 5′-3′ XRN1-mediated, RNA degradation is required for the anti-JEV activity of ZAP. A549 cells with shRNA targeting LacZ, XRN1, and EXOSC5 were transduced by lentiviruses expressing EGFP, ZAP-L-V5, and ZAP-S-V5. After 10 h of JEV infection (MOI = 5), cells lysates were analyzed by western blot for the indicated proteins (A and D, upper panel). The relative quantification of NS3 normalized by actin was quantified by ImageJ software (A and D, lower panel). Data are mean ± SD (from four independent experiments). Total RNA and culture supernatants were harvested for the measurement of viral RNA by RT-PCR (B and E) and viral titer by plaque assay (C and F). Data are representative results from repeated experiments and shown as mean ± SD (n = 3). The statistical significance was estimated by two-tailed Student’s *t* test. * *P*≤0.05; ** *P*≤0.01; *** *P*≤0.001; *NS*, not significant.

ZAP modulates target RNA by recruiting cellular 5′-3′ XRN1-dependent and 3′-5′ RNA exosome-dependent RNA decay machineries [6, 10]. To evaluate whether cellular RNA decay machineries participated in the anti-JEV activity of ZAP, we depleted the expression of XRN1 or EXOSC5 in ZAP-L, ZAP-S and EGFP-overexpressing cells by using a shRNA-targeting approach. Compared with the shLacZ control, knockdown of XRN1 did not affect the anti-JEV activity of ZAP (Fig 5A–5C), whereas the anti-JEV effect of ZAP was hampered by knockdown of EXOSC5 (Fig 5D–5F). The data suggest that 3′-5′ RNA degradation by the exosome complex is involved in the antiviral mechanism of ZAP against JEV infection.

### ZAP enhances innate immune responses through the RIG-I signaling pathway

**Fig 6:**
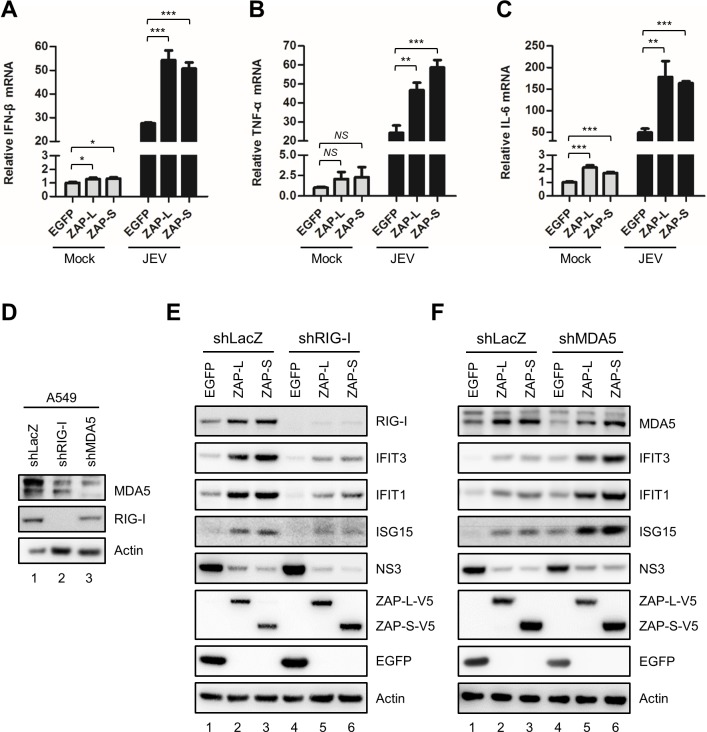
ZAP-enhanced innate immune responses in JEV-infected cells. (A-C) A549 cells overexpressing EGFP, ZAP-L and ZAP-S were uninfected (Mock) or infected with JEV (MOI = 5) for 16 h. Total RNA was collected for IFN-β, TNF-α, and IL-6 mRNA detection by RT-qPCR. RT-qPCR results are presented relative to the expression of GAPDH. Representative data from repeated experiments with triplicate samples (mean ± SD, n = 3) were analyzed by two-tailed Student’s *t* test. * *P*≤0.05; ** *P*≤0.01; *** *P*≤0.001. (D) Western blot analysis for the knockdown effects of RIG-I and MDA5 in A549-shLacZ, -shRIG-I and -shMDA5 cells established by transduction with lentiviruses expressing the indicated shRNA. (E and F) A549-shLacZ, -shRIG-I and -shMDA5 cells transduced by lentiviruses expressing EGFP, ZAP-L-V5, and ZAP-S-V5 were infected with JEV (MOI = 5) for 16 h. Western blots analysis was performed for the indicated proteins.

To address whether ZAP boosted innate immune responses during JEV infection, we measured the mRNA levels of type I IFN and proinflammatory cytokines in A549 cells with or without ZAP overexpression. As compared with the EGFP control, ZAP-L and ZAP-S slightly increased the basal level of IFN-β, TNF-α and IL-6 (Fig 6A–6C), as well as chemokines CXCL10 and CCL5 (S8 Fig) in uninfected cells as previously reported [13], and further increased the expression of these cytokines/chemokines in JEV-infected A549 cells (Fig 6A–6C, S8 Fig). We further determined the upstream RLRs involved in the sensing events by knocking down the expression of RIG-I or MDA5 by lentiviral transduction (Fig 6D). Compared with the shLacZ control, knockdown of RIG-I, but not MDA-5, reduced the induction of interferon-stimulated genes (ISGs), e.g., IFIT1, IFIT3, and ISG15 that was seen in ZAP overexpressing cells (Fig 6E and 6F), indicating that ZAP-enhanced innate immune responses mainly went through the RIG-I signaling pathway in JEV-infected cells. Interestingly, knockdown of MDA5 even elevated the induction of ISGs (Fig 6F), similarly to a previous report showing higher IFN-β production in *MDA5*^-/-^ but not *RIG*^-/-^ mouse embryonic fibroblasts infected with JEV [26]. Thus, deprive of MDA5 in JEV-infected cells might trigger some compensational IFN-related signaling events through unclear mechanism. Moreover, the anti-JEV effect of ZAP measured by viral NS3 protein expression was still noted in cells deprived of RIG-I with decreased ISGs expression (Fig 6E) as well as in the type I IFN-deficient Vero cells with ectopic ZAP expression (S9 Fig). Thus, ZAP-enhanced innate immune response was not involved in the anti-JEV effect, probably because JEV can block the JAK-STAT pathway and is somewhat resistant to type I IFN [27, 28].

### ZAP targets the 3′-UTR of JEV

**Fig 7:**
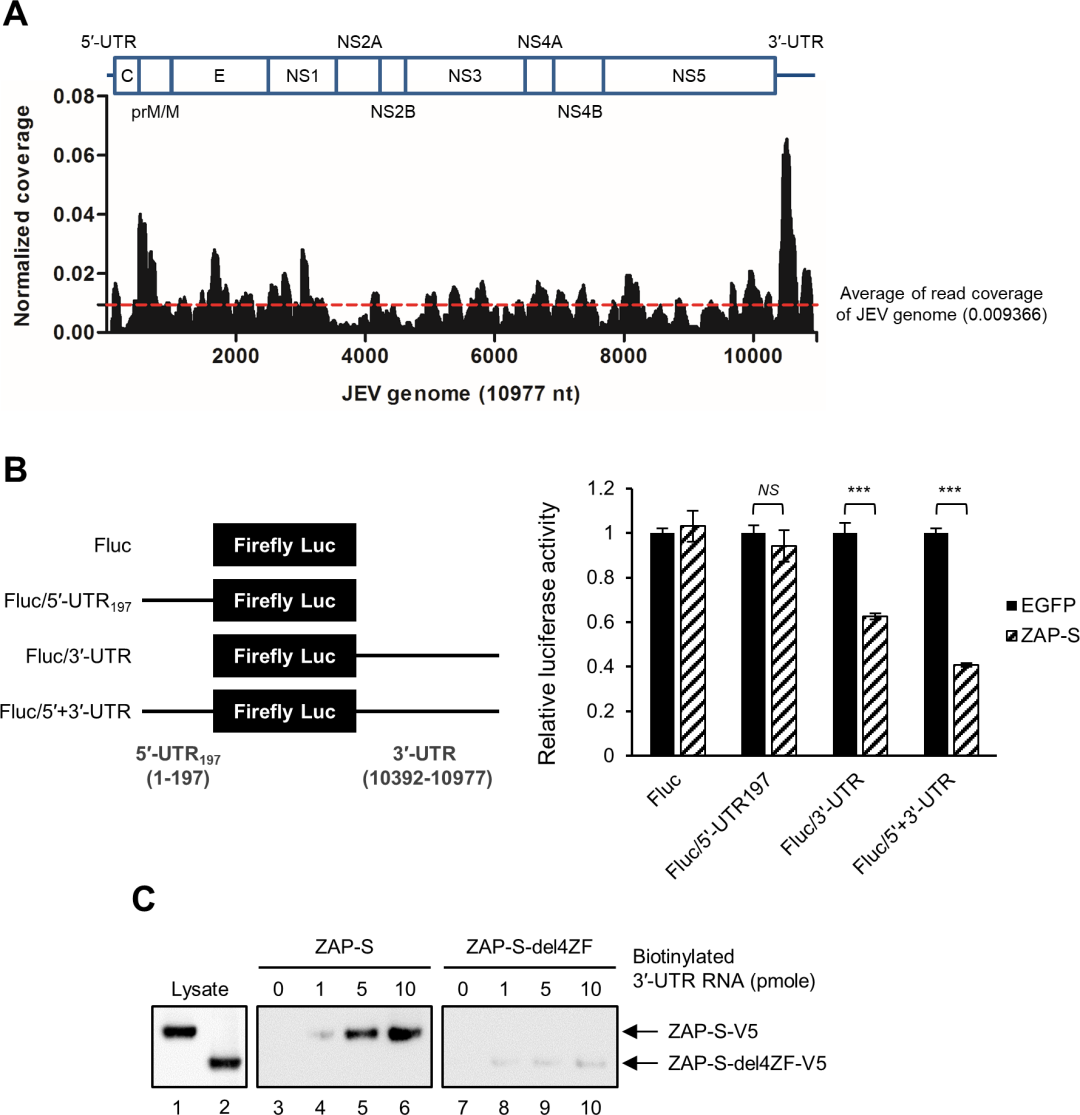
ZAP mainly targets JEV 3′-UTR. (A) Map of ZAP-S binding sites in full-length JEV genome by CLIP-seq of RNA isolated from JEV-infected ZAP-S overexpressing A549 cells. Read coverage, the reads of each position normalized to the total number of reads mapping to the viral genome. (B) Schematic diagram of the reporter RNAs (left panel). 293T/17-EGFP and -ZAP-S cells were cotransfected with 5′-capped firefly luciferase (Fluc) flanked by JEV 5′-UTR_197_ and/or 3′-UTR RNA and control Renilla luciferase (Rluc) RNA for 18 h. Relative luciferase activity was measured by dual-luciferase reporter assay (right panel). Representative data from two independent experiments are mean ± SD (n = 3) and analyzed by two-tailed Student’s *t* test. *** *P*≤0.001; *NS*, not significant. (C) Biotin-labeled JEV 3′-UTR RNA (at the indicated amounts) was incubated with ZAP-S or ZAP-S-del4ZF overexpressing A549 cell extracts and then pulled down by using streptavidin beads. The co-precipitated ZAP-S-V5 (WT and del4ZFs) was assayed by western blot.

Both RNA sequence and structure have been considered as important for ZAP recognition [29, 30]; however, the common features of ZAP-responsive elements (ZRE) are still inconclusive. To elucidate the possible regions of JEV genome targeted by ZAP, we performed UV crosslinking and immunoprecipitation (CLIP) to pull down the RNA interacting with ZAP in JEV-infected ZAP-S overexpressing A549 cells. The enriched RNA population was subjected to next generation sequencing (NGS) by use of the Ion Torrent platform. The read coverages of several peaks/regions were above the average read coverage especially within the JEV 3′-UTR (Fig 7A). We thus focused on 5′- and 3′-UTR, which contain conserved complementary sequences and extensive secondary structures to regulate viral translation and transcription [15, 16], to elucidate the potential ZRE in the JEV genome. We designed reporter constructs containing the 5′ 197 nt. (5′-UTR plus the first 102 nt. of the core gene) and/or the entire 3′-UTR flanking firefly luciferase (Fluc) in the pGL3-promoter vector under a SP6 promoter (Fig 7B, left panel). The *in vitro* transcribed reporter RNA and control Renilla luciferase (Rluc) RNA were cotransfected into 293T/17 cells with or without ZAP-S overexpression. As compared with the EGFP control, ZAP did not influence the luciferase activity of the reporter RNA with 5′-UTR_197_, while ZAP significantly reduced those with 3′-UTR (Fig 7B, right panel), revealing the possible ZRE in the JEV 3′-UTR. *In vitro* RNA pull-down assay also confirmed that the interaction between ZAP-S and JEV 3′-UTR in a ZF domain dependent manner (Fig 7C) was stronger than that with 5′-UTR_197_ (S10 Fig).

### Mapping the ZRE in JEV 3′-UTR

**Fig 8:**
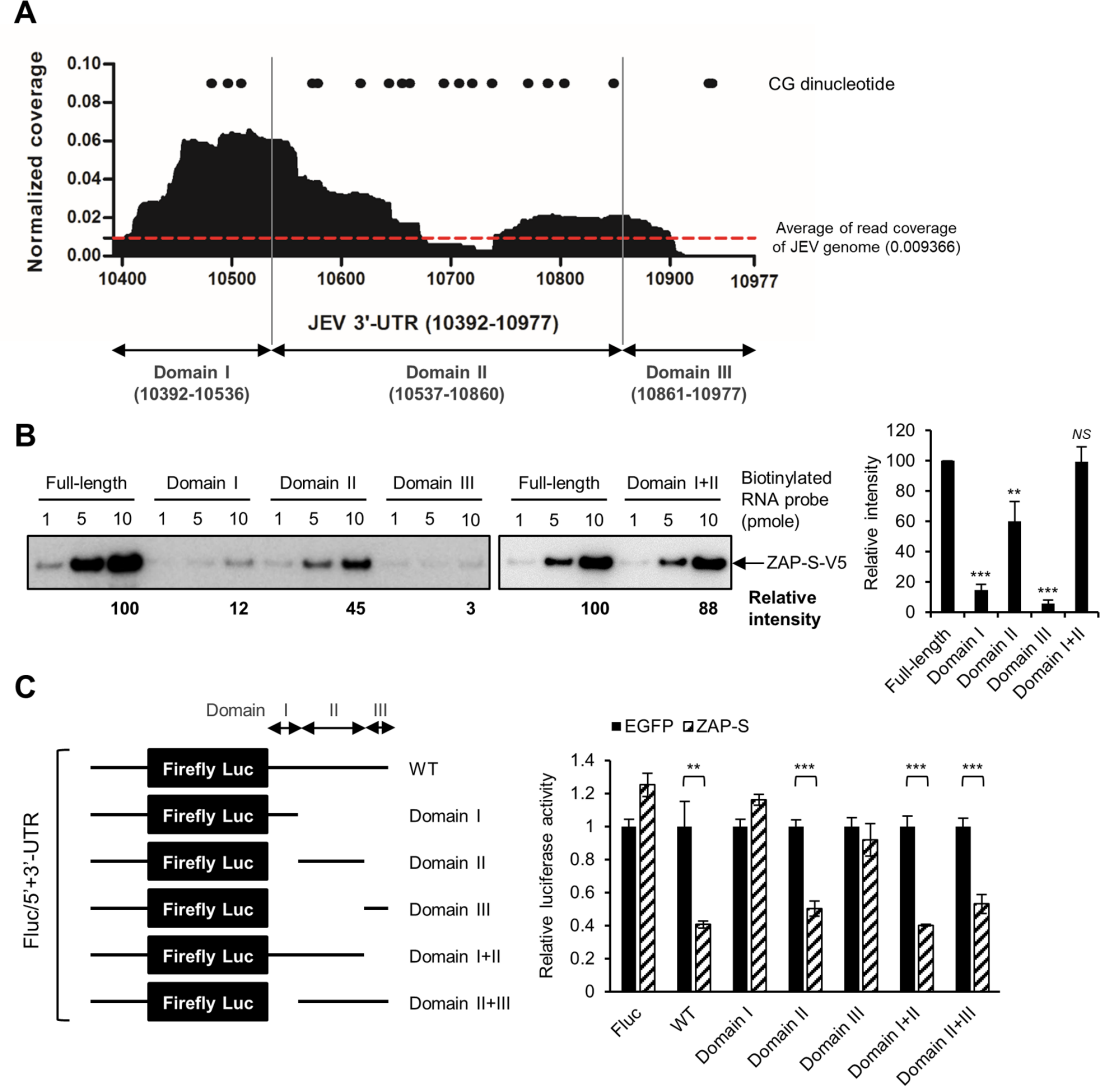
Mapping of ZAP-responsive element (ZRE) in JEV 3′-UTR. (A) The CLIP-seq diagram of ZAP-S binding RNA mapped to JEV 3′-UTR. Read coverage, the reads of each position normalized to the total number of reads mapping to the viral genome. The JEV 3′-UTR is divided into domain I, II and III as indicated. The positions of CG dinucleotide are shown by dots. (B) Biotin-labeled JEV 3′-UTR RNA probes (full-length and individual domain I, II, III, and I+II) incubated with A549-ZAP-S cell extracts were pulled down with streptavidin beads and determined for ZAP-S-V5 protein by western blot (left panel). The intensity of ZAP-S was quantified by ImageJ software. Mean ± SD was calculated from 3 independent experiments and analyzed by two-tailed Student’s *t* test (right panel). (C) 5′-capped full-length and deleted Fluc/5′+3′-UTR RNA (left panel) and control Rluc RNA were cotransfected into 293T/17-EGFP and -ZAP-S cells. At 18 h post-transfection, cell lysates were collected to perform dual-luciferase assay (right panel). Representative data from three independent experiments shown as mean ± SD (n = 3) were analyzed by two-tailed Student’s *t* test. ** *P*≤0.01; *** *P*≤0.001; *NS*, not significant.

To further evaluate the possible ZRE in the 3′-UTR of JEV genome, CLIP-seq results within the three defined domains of JEV 3′-UTR: domain I (variable region), domain II (dumbbell structure) and domain III (3′ conserved sequence and terminal stem-loop) [31] are shown (Fig 8A). We measured the binding ability of these RNA domains with ZAP by using biotinylated RNA. The proteins pulled down by streptavidin were subjected to western blotting with anti-V5 antibody for ZAP-S level. Domain II (showing a 45% binding ability of the full-length) had the strongest interaction with ZAP when compared to domain I (12% of the full-length) and domain III (3% of the full-length) of the JEV 3′-UTR RNA (Fig 8B). Interestingly, domain II contained high frequency of CG dinucleotide (Fig 8A), which was recently reported to confer ZAP binding and recognition [32]. Furthermore, the binding of ZAP to domain I+II RNA containing most of the CG dinucleotides reached 88% of the full length 3′-UTR RNA (Fig 8B). To verify the regions targeted by ZAP, we generated five reporter RNAs containing domain I, II, III, I+II, and II+III, respectively (Fig 8C, left panel). Significant reduction of luciferase activity by ZAP-S was noted in the reporters with WT, domain II, domain I+II and domain II+III, indicating the importance of domain II in conferring the sensitivity to ZAP (Fig 8C, right panel). Thus, domain II of JEV 3′-UTR containing dumbbell structures and high content of CG dinucleotide may function as ZRE and contribute to ZAP sensitivity.

## Discussion

ZAP exhibits antiviral activity against a variety of viruses, but flaviviruses were not known to be sensitive to ZAP until this study. Here, we demonstrate that overexpression of human ZAP (isoforms ZAP-L and ZAP-S) inhibited JEV infection and downregulation of endogenous ZAP enhanced JEV replication, indicating the intrinsic antiviral potential of ZAP. We also found that, similar to YFV [4], DENV and ZIKV are resistant to ZAP, supporting the notion that ZAP is not a universal antiviral factor even for viruses within the same family.

Viruses may use different strategies to evade the ZAP-mediated antiviral action, but it is still not fully understood what determines the sensitivity of a virus to ZAP. Influenza A virus NS1 protein antagonizes ZAP-S by interrupting its binding to target mRNA [19]. Murine gammaherpesvirus 68 RTA inhibits the antiviral activity of ZAP by disrupting the N-terminal intermolecular interaction of ZAP [20]. HSV-1 UL41 endoribonuclease was identified as an antagonist of human ZAP by degrading its mRNA [17]. Enterovirus 71 3C protease mediates the cleavage of ZAP protein [18]. In our hands, DENV neither downregulated the expression of ZAP nor blocked the antiviral activity of ZAP (S4 and S5 Figs). However, we were able to detect the interaction of ZAP with JEV RNA (Figs 2F and 3B) but not with DENV RNA (S7 Fig), consistent with the antiviral potential of ZAP against JEV but not DENV. Thus, the resistance of DENV to ZAP might be resulted from a failure of ZAP to bind with DENV viral RNA.

Recently, viral RNA with high CG dinucleotide content was found to be targeted by ZAP, and ZAP bound directly and selectively to RNA sequences containing CG dinucleotides [32]. The known ZAP-sensitive virus genomes contained relatively higher CG frequencies, such as ZAP-sensitive SINV (0.9) *vs*. ZAP-resistant YFV (0.38) [32]. Interestingly, the ZAP-sensitive JEV genome also has a relatively higher CG frequency (0.61) as compared to that of ZAP-resistant ZIKV (0.46) and DENV (0.36). Moreover, we detected the interaction of ZAP with JEV RNA (Figs 2F and 3B) but not with DENV RNA (S7 Fig). Thus, JEV RNA enriched with CG dinucleotides may be targeted by ZAP binding to trigger the antiviral effect. Our crosslinking-immunoprecipitation-sequencing assay showed several peaks of ZAP binding in the JEV 3′-UTR, and domain II of JEV 3′-UTR with high frequencies of CG dinucleotide was further mapped as the major ZRE (Figs 7 and 8). Since domain II also contains stem-loop and pseudoknot structures [31, 33], the contribution of RNA secondary structures in addition to CG dinucleotides in ZAP recognition cannot be ruled out.

ZAP-S serves as a key regulator of RIG-I-mediated innate immune responses for type I IFN production to limit the infection of influenza A virus and Newcastle disease virus. Besides, ZAP-S also enhances NF-κB and IRF3 signaling downstream of RIG-I for the induction of proinflammatory cytokines, like TNF-α and IL-6 [13]. In this study, we showed that ZAP promoted the production of IFN-β, TNF-α and IL-6 during JEV infection (Fig 6A–6C). It has been speculated that elevated IFNs, TNF-α, and IL-6 during viral infection correlated with the severity and outcome of viral diseases. Similarly, IFNs and proinflammatory cytokines including TNF-α and IL-6 were elevated in patients with acute Japanese encephalitis [34–36]. Thus, the induction of IFN and proinflammatory cytokines/chemokines by ZAP may contribute to host defense responses as well as the JEV-induced inflammatory states and pathogenesis during JEV infection.

ZAP restricted JEV infection mainly by posttranscriptional regulation such as blocking protein translation and enhancing RNA degradation. Through interaction with XRN1 and exosome components, like Rrp46 (EXOSC5), Rrp40 (EXOSC3) and Rrp42 (EXOSC7) [6, 10], ZAP utilizes cellular RNA decay machineries to destabilize both viral and cellular RNAs. ZAP binds with 5′-UTR of HIV-1 *nef* mRNA and 3′-UTR of cellular TRAILR4 mRNA and then requires RNA exosome, and potentially XRN1, to degrade the target RNAs [6, 25]. The anti-JEV effect of ZAP was diminished by knockdown of the exosome component (Fig 5D–5F), indicating the involvement of 3′-5′ RNA decay in the ZAP antiviral pathway. Thus, ZAP can bind with JEV RNA and target the viral RNA to the 3′-5′ RNA exosome complex for RNA degradation. Furthermore, XRN1-dependent RNA decay is known to generate subgenomic flavivirus RNA (sfRNA) [37, 38], which can then block XRN1 activity and alter host mRNA stability [39]. Interestingly, XRN1 was not involved in the anti-JEV activity of ZAP (Fig 5A–5C), probably due to the interplay between XRN1 and sfRNA in JEV-infected cells prevailing the involvement of XRN1 in the antiviral action of ZAP. Since ZAP not only restricts viral infection but also regulates cellular mRNA abundance [25], we cannot exclude the possibility that the blocking effect of ZAP on JEV is due to the altered cellular mRNA and protein expression. Overall, we have identified JEV as the first flavivirus sensitive to human ZAP and provide insight about the antiviral mechanism of ZAP against viral RNA without 3′-poly(A) tail.

## Materials and methods

### Viruses, cell lines and chemicals

Neurovirulent JEV RP-9 strain plaque-purified from a Taiwanese NT109 isolate (GenBank accession: AF014161) [40], DENV-2 PL046 strain (GenBank accession: KJ734727) [41] and ZIKV PRVABC59 strain (2015 Puerto Rico strain, GenBank accession: KU501215) were propagated in mosquito C6/36 cells (ATCC, CRL-1660) maintained in RPMI-1640 medium supplemented with 5% fetal bovine serum (FBS). Recombinant Sindbis virus containing a *Firefly luciferase* reporter gene (dSinF-Luc/2A) [42] was amplified in baby hamster kidney cells (BHK-21). BHK-21 cells (ATCC, CCL-10) for JEV and DENV titration were grown in RPMI-1640 medium supplemented with 5% FBS, 2 mM L-glutamine and 1% penicillin-streptomycin (P/S). African green monkey kidney Vero cells (ATCC, CCL-81) for ZIKV titration were grown in Minimum Essential Medium (MEM) supplemented with 10% FBS, 2 mM L-glutamine and 1% P/S. Human lung carcinoma A549 cells (ATCC, CCL-185) were cultured in F-12 medium containing 10% FBS, 2 mM L-glutamine and 1% P/S. Human embryonic kidney 293T/17 cells (ATCC, CRL-11268) were cultured in Dulbecco’s Modified Eagle’s Medium (DMEM) containing 10% FBS and 2 mM L-glutamine. Human neuroblastoma BE(2)C cells (ATCC, CRL-2268) were cultured in RPMI-1640 medium containing 10% FBS, 2 mM L-glutamine and 1% P/S. Human neuroblastoma SK-N-SH cells (ATCC, HTB-11) and human microglia HMC3 cells (ATCC, CRL-304) were grown in MEM supplemented with 10% FBS, 2 mM L-glutamine and 1% P/S. Human NPCs (hNPCs) were generated from human skin fibroblast reprogrammed hiPSCs [43] from healthy donor following the procedure described previously [44]. The hNPCs were maintained on MatrigelTM-coated dishes and cultured in the neural induction medium: DMEM/F12 with MEM-NEAA (1:100, Invitrogen), N2 supplement (1:100, Invitrogen), B27 supplement (1:50, Invitrogen), Heparin (2 mg/mL, Sigma-Aldrich), cAMP (1 μM, Sigma-Aldrich) and IGF-1 (10 ng/ml, Peprotech). Lipofectamine 2000 reagent (Invitrogen) was used for DNA and RNA transfection.

### Preparation of lentiviral vectors

293T/17 cells were cotransfected with shRNA expressing pLKO.1 lentiviral construct or protein expressing pSIN lentiviral construct plus 2 helper plasmids, pMD.G and pCMVΔR8.91, using Lipofectamine 2000. For shRNA expressing lentiviruses, culture supernatants containing lentiviral particles were harvested and stored at 80°C. For protein expressing lentiviruses, culture supernatants were further concentrated by Speedy Lentivirus Purification kit (LV999, abm). After lentiviral transduction, cells were selected by 10 μg/mL puromycin (InvivoGen) for stable cell line establishment.

### Plasmid constructs

The cDNA of human ZAP-L (NM_020119) was amplified from interferon (IFN)-treated A549 cells with the primer sequences 5′-CGCCATGGCGGACCCGGAGGTG-3′ and 5′-ACTAATCACGCAGGCTTTGTCTTCAGT-3′, and cloned into pcDNA3.1/V5-His vector by TOPO-TA cloning (K4800, Invitrogen). Human ZAP-S (NM_024625) construct was generated by mutating nt. 2097 and removing nt. 2098–2706 of ZAP-L coding sequences in ZAP-L/pcDNA3.1/V5-His with the primer sequences 5′-CAGATGAAGAGAGGGCCAGAGAAGGGCAATTCTGCAGATATC-3′ (mutation underlined) by single-primer mutagenesis [45]. The portions of ZAP-L and ZAP-S fused with V5-tag were further subcloned to the self-inactivating lentiviral vector (pSIN), in which the expression of an inserted gene is under the control of a constitutive spleen focus-forming virus (SFFV) promoter [46]. The zinc-finger domains deleted ZAP-L and ZAP-S were generated by single-primer mutagenesis with the ZAP-del4ZFs (73–193) primer (5′-ACCACTCGAGCCCGGGTC/ATGGACAGAAAGGTGCTGGCCATCA-3′). JEV 5′+3′-UTR/pGL3-promoter plasmid consisted of a SV40 promoter upstream of a firefly luciferase (Fluc) gene flanked by the 5′-UTR plus partial core gene (nt. 1–197) at its 5′-end and the 3′-UTR (nt. 10392–10977) at its 3′-end. The cDNAs of nt. 1–197 and nt. 10392–10977 were amplified and inserted into the 5′ (HindIII/NcoI sites) and 3′ (XbaI site) of the firefly luciferase gene in pGL3-promoter vector (Promega), respectively. The two 3′-UTR deleted constructs: 5′+3′-UTR/domain II+III and 5′+3′-UTR/domain III, were generated by removing nt. 10392–10536 and nt. 10392–10860 of 3′-UTR. Lentiviral vectors containing shRNA targeting human ZC3HAV1 (5′-GTAAGGGTTGTCCGCTTAATG-3′, TRCN0000419627), human XRN1 (5′-GTTACTCACAGGTCGTAAATA-3′, TRCN0000296739), human EXOSC5 (5′-GTGAAGGTCAGCAAAGAGATT-3′, TRCN0000050621), human RIG-I (5′-AGCACTTGTGGACGCTTTAAA-3′, TRCN0000230212), human MDA5 (5′-CCAACAAAGAAGCAGTGTATA-3′, TRCN0000050849) and LacZ (5′-CGCGATCGTAATCACCCGAGT-3′, TRCN0000072224) were obtained from the National RNAi Core Facility, Taiwan.

### Antibodies

Anti-JEV NS3 and anti-DENV NS3 antibodies were described previously [41, 47]. The following commercial antibodies were used: mouse anti-V5 (V8012) and rabbit anti-V5 (V8137) from Sigma-Aldrich; anti-ZC3HAV1 (ZAP) (GTX120134), anti-IFIT1 (GTX103452), and anti-IFIT3 (GTX112442) from GeneTex; anti-actin (NB600-501) and anti-XRN1 (NB500-191) from Novus Biologicals; anti-RIG-I (#3743) and anti-ISG15 (#2743) from Cell Signaling Technology; anti-MDA5 (ALX-210-935-C100) from Enzo Life Sciences; anti-EXOSC5 (ab168804) from Abcam; and anti-GFP (11814460001) from Roche.

### Reverse transcription, RT-PCR and real-time quantitative PCR (RT-qPCR)

Total RNA was extracted using RNeasy Mini Kit (Qiagen). For RT-PCR, 1 μg of RNA was reverse-transcribed by random hexamer primer with SuperScript III First-Strand Synthesis System (Invitrogen). PCR was then performed by using the primers described in S1 Table. For real-time qPCR, random hexamer primer was used for reverse transcription and qPCR was performed by ABI-Prism 7500 real-time PCR system (Applied Biosystems). The relative RNA levels of specific RNA were normalized with GAPDH or firefly luciferase, and calculated by the comparative threshold cycle (ΔΔCt) method. The TaqMan Universal PCR Master Mix with UNG (Invitrogen) and commercial probes for human GAPDH (Hs02758991), IFN-β (Hs01077958), IL-6 (Hs00985639), TNF-α (Hs01113624), CCL5 (Hs00982282), CXCL10 (Hs01124251) and firefly luciferase (Mr03987587) (Applied Biosystems) were used in qPCR reactions. The JEV viral RNA primers for qPCR have been described previously [48].

### Immunofluorescence analysis

Cells were fixed by 4% paraformaldehyde in PBS for 20 min and permeabilized by 0.1% TritonX-100 in PBS for 5 min. After blocking with PBS containing 3% bovine serum albumin (BSA), cells were incubated with primary antibodies against dsRNA (1:1000, J2, Scicons) and ZAP-L/S (1:100, 16820-1-AP, Proteintech) in TBS containing 1% BSA at 4°C overnight. After washing with TBS, Alexa Fluor conjugated secondary antibodies (Alexa Fluor 488 goat anti-mouse IgG [A11001] and Alexa Fluor 568 goat anti-rabbit IgG [A11036], Molecular Probes) for respective dsRNA and ZAP-L/S detection were added for 2 h at room temperature. Cell nuclei were stained with 4′,6′-diamidino-2-phenylindole (DAPI) (1:2000, Molecular Probes). Cells were photographed under a Zeiss LSM700 Meta Confocal Microscope with a 63X objective. Co-localization was calculated by Pearson’s correlation coefficient (PCC) using ImageJ software (NIH).

### RNA immunoprecipitation (RIP) assay

Co-immunoprecipitation and RT-PCR were combined to examine the association of viral RNA with ZAP proteins. Cells were lysed by RIPA lysis buffer (10 mM Tris-HCl [pH 7.5], 150 mM NaCl, 5 mM EDTA, 0.1% SDS, 1% Triton X-100, and 1% sodium deoxycholate) containing protease inhibitor cocktail and RNase inhibitor. For RIP assay using antibody against endogenous ZAP-L/S, mock and virus-infected A549 cell extracts were immunoprecipitated with preincubated mixture of protein A (Protein A Mag Sepharose Xtra, 28-9670-62, GE Healthcare) with anti-ZC3HAV1 (ZAP) antibody (16820-1-AP, Proteintech) or IgG (12–370, EMD Millipore) at 4°C for 4 h. For RIP assay using anti-V5 antibody, extracts of virus-infected 293T/17 cells expressing EGFP (without V5-tag), ZAP-L-V5, and ZAP-S-V5 (WT and del4zFs) were incubated with anti-V5 agarose affinity gel (A7345, Sigma-Aldrich) at 4°C overnight. The antibody-protein-RNA complex was then washed five times by RIPA lysis buffer, and the bound RNA was purified by RNeasy Mini Kit (Qiagen). RT-PCR was performed by use of the 3′-UTR specific primers (S1 Table).

### *In vitro* transcription

The RdRP-dead JEV replicon DNA was amplified from SP6-JR2A NS5mt/pBR22 plasmid [24]. The DNA fragments of Fluc/5′-UTR_197_, Fluc/3′-UTR, and Fluc/5′+3′-UTR for *in vitro* RNA transcription were amplified from JEV 5′+3′-UTR/pGL3-promoter plasmid. The DNA fragments of domain I, domain II, domain III, domain I+II, and domain II+III Fluc/5′+3′-UTR were amplified by using the primers shown in S2 Table. RNA transcripts were *in vitro* synthesized using RiboMAX Large Scale RNA Production System-SP6 (Promega), and the cap analog [m7G(5')ppp(5')G] (Ambion) was used for 5′-capped RNA synthesis. JEV 5′-UTR_197_ and 3′-UTR (full length, domain I, domain II, domain III, and domain I+II) DNA templates for *in vitro* transcription of biotinylated RNA probes were amplified from JEV 5′+3′-UTR/pGL3-promoter plasmid. Biotin-labeled RNAs were synthesized by adding additional 2.5 μL of 10 mM biotin-16-UTP (Roche) into a 50 μL *in vitro* transcription mixture. After incubation at 37°C for 3 h, template DNA was removed with RNase-free DNase I. Synthesized RNAs were purified by Direct-zol RNA kit (Zymo Research) and quality checked by agarose electrophoresis. The primer sets for the specific PCR products above are described in S2 Table.

### *In vitro* RNA pull-down assay

Different amounts (0, 1, 5, and 10 pmole) of biotin-labeled RNA probe were incubated with 100 μg of ZAP-S-V5 or ZAP-S-del4ZFs expressing A549 cell extracts lysed by CHAPS lysis buffer (10 mM Tris-HCl [pH 7.4], 1 mM MgCl_2_, 1 mM EGTA, 0.5% CHAPS, 10% glycerol, and 5 mM 2-mercaptoethanol) containing protease inhibitor cocktail in a final volume of 100 μl of RNA binding buffer (10 mM Tris-HCl [pH 7.5], 50 mM NaCl, 1 mM EDTA, and 10 μM ZnCl_2_) supplemented with 1 unit/μl RNasin (Promega), 1 μg/μl heparin (Sigma-Aldrich) and 100 ng/μl yeast tRNA (Roche) for 30 min at 30°C. After incubation, 300 μl of Streptavidin MagneSphere paramagnetic particles (Promega) was added for 30 min at 25°C. Protein-RNA complexes were washed three times by RNA binding buffer without heparin and yeast tRNA. After washing, 30 μl of 2X SDS-PAGE sample buffer was added and incubated for 10 min at room temperature. The pull-down proteins were further analyzed by western blot.

### Measurement of replication-defective replicon RNA stability

5′-capped Renilla luciferase containing JR2A-NS5mt replicon RNA and control firefly luciferase RNA generated by *in vitro* transcription were cotransfected into EGFP, ZAP-L, and ZAP-S overexpressing 293T/17 cells for 3 and 9 h. Total RNA was collected for detection of incoming RNA by RT-qPCR. The relative JEV replicon RNA levels were normalized with that of firefly luciferase.

### Reporter assay

5′-capped firefly luciferase containing reporter RNA and control Renilla luciferase RNA by *in vitro* transcription were cotransfected into EGFP and ZAP-S overexpressing 293T/17 cells. The relative luciferase activity was assessed by dual-luciferase reporter assay system.

### Cross-linked immunoprecipitation followed by next generation sequencing (CLIP-seq)

CLIP assay was performed based on a previously published protocol [49]. A549-ZAP-S-V5 cells infected with JEV (MOI = 5) for 24 h were UV-crosslinked at 254 nm with 200 mJ/cm^2^ using UV Stratalinker 2400 (Stratagene). Cells were lysed with CLIP lysis buffer containing protease inhibitor cocktail (50 mM Tris-HCl [pH7.4], 100 mM NaCl, 1% NP-40, 0.1% SDS, and 0.5% sodium deoxycholate), treated with 10 μL of RQ1 DNase (Promega) and 10 μL of RNase A/T1 (1:500 dilution) (EN0551, Thermo Fisher Scientific) on a shaker at 1000 rpm for 5 min at 37°C. After treatment, cell lysates were immunoprecipitated with preincubated anti-V5 antibody-protein G complex (Anti-V5 antibody, R960-25, Invitrogen; Protein G Mag Sepharose Xtra, 28-9670-70, GE Healthcare) for 4 h at 4°C. Samples were then washed with CLIP lysis buffer containing 500 mM NaCl and CLIP lysis buffer twice, respectively. Protease K (4 mg/mL) in protease K buffer (100 mM Tris-HCl [pH7.4], 50 mM NaCl, and 10 mM EDTA) was preincubated for 20 min at 37°C to ensure digestion of RNase. The RNA-protein-antibody immune complexes were incubated on a shaker at 1000 rpm for 20 min at 37°C. After digestion by protease K, the bound RNA was purified by Direct-zol RNA kit (Zymo Research). The sequence library was constructed using Ion Total RNA-seq Kit v.2 (Thermo Fisher Scientific) and the library was sequenced by Ion Torrent PGM system (Thermo Fisher Scientific). Downstream data analysis was performed by use of the CLC Genomics Workbench 11.0.1 (Qiagen). The alignment of trimmed reads to JEV RP-9 reference sequence (GenBank accession: AF014161) was based on the following parameters: match score = 1; mismatch cost = 2; insertion/deletion cost = 3; length fraction = 0.9; similarity fraction = 0.9; Non-specific match handling = ignore. Read coverage showed the read numbers of each position mapping to JEV genome divided by the total read numbers mapping to JEV genome. The CLIP-seq data have been deposited in the NCBI GEO data repository with accession code GSE115747.

### Statistical analysis

Two-way AVONA was used to estimate the statistical significance of viral replication kinetics data. Two-tailed Student’s *t* test was used to estimate the statistical significance between two groups. Representative data from repeated independent experiments are shown as mean ± standard deviation (SD) with triplicate samples (n = 3). *P*≤0.05 was considered statistically significant. * *P*≤0.05; ** *P*≤0.01; *** *P*≤0.001; *NS*, not significant.

## Supporting information

S1 Fig ZAP exerts anti-JEV activity in human neuronal cells. Human neuronal hNPCs (A), HMC3 cells (B), BE(2)C cells (C), and SK-N-SH cells (D) transduced with lentiviruses expressing EGFP, ZAP-L-V5, and ZAP-S-V5 were infected by JEV (MOI = 1 for hNPCs and MOI = 5 for the others) for 16 h. Cell lysates and culture supernatants were harvested to determine protein level (left panel) and viral titer (right panel) by western blot and plaque assay, respectively. Viral titration data are mean with individual data from 2 independent experiments.
(TIF)

S2 Fig Antiviral potential of ZAP against ZIKV infection. A549 cells expressing EGFP, ZAP-L-V5, and ZAP-S-V5 by lentiviral transduction were infected with ZIKV (MOI = 5) for 10 and 24 h. Collected cell lysates and culture supernatants were used to perform western blot (A) and plaque assay (B). Viral titers are mean with individual data from 2 independent experiments.
(TIF)

S3 Fig ZAP does not block low MOI of DENV and ZIKV infection. A549-EGFP, ZAP-L, and ZAP-S cells were infected with DENV (A) and ZIKV (B) (MOI = 0.1) for the indicated hours. Cell lysates were assayed for the indicated proteins by western blot.
(TIF)

S4 Fig Endogenous level of ZAP proteins during JEV and DENV infection. Western blot analysis for the indicated proteins in mock and JEV (A) or DENV (B) (MOI = 10) infected A549 cells at the indicated times post-infection.
(TIF)

S5 Fig DENV does not interfere with the antiviral activity of ZAP against SINV. A549-EGFP and -ZAP-S cells were mock-infected or infected with DENV (MOI = 5) for 2 h, followed by infection of Sindbis virus expressing firefly luciferase (dSinF-Luc/2A) (MOI = 5) for additional 24 h. The infection of dSinF-Luc/2A was assessed by firefly luciferase assay. Data are mean ± SD (n = 3) and analyzed by two-tailed Student’s *t* test. *NS*, not significant.
(TIF)

S6 Fig Antiviral potential of endogenous ZAP in BE(2)C cells against JEV infection. (A) Western blot analysis of ZAP isoforms and actin in BE(2)C cells with shLacZ or shZAP-L/S knockdown. (B and C) Viral RNA (B) and viral titer (C) analysis of JEV (MOI = 5) infected BE(2)C cells with shLacZ or shZAP-L/S after 12 and 24 h of infection. Relative JEV RNA level normalized by GAPDH was determined by using RT-qPCR. Viral titer was determined by plaque assay. Data are mean ± SD (n = 3) and analyzed by two-tailed Student’s *t* test. * *P*≤0.05; ** *P*≤0.01.
(TIF)

S7 Fig RNA-binding activity of ZAP to DENV RNA. 293T/17 cells transfected with plasmids expressing EGFP, ZAP-L-V5, and ZAP-S-V5 for 24 h were infected with DENV (MOI = 10) for additional 6 h. Cell lysates were subjected to immunoprecipitation by anti-V5 agarose affinity gel, and the pull-down ZAP proteins were detected by western blot with anti-V5 antibody (upper panel). DENV RNA and cellular TRAILR4 mRNA were amplified by RT-PCR using DENV 3′-UTR and TRAILR4 specific primers (the second and third panel). RT-PCR of input viral RNA in DENV-infected cells by detection of 3′-UTR (lower panel).
(TIF)

S8 Fig ZAP enhances chemokine expression in JEV-infected cells. Total RNA were harvested from uninfected (Mock) and JEV (MOI = 5) infected A549-EGFP, -ZAP-L, and -ZAP-S cells at 16 hpi. The relative RNA levels of CXCL10 (A) and CCL5 (B) normalized by GAPDH were analyzed by RT-qPCR. Data are mean ± SD (n = 3) and analyzed by two-tailed Student’s *t* test. * *P*≤0.05; ** *P*≤0.01; *** *P*≤0.001; *NS*, not significant.
(TIF)

S9 Fig ZAP shows anti-JEV activity in Vero cells. Vero-EGFP, -ZAP-L, and -ZAP-S cells established by lentiviral transduction were infected with JEV (MOI = 5) for 16 and 24 h. Western blot analysis for the indicated proteins. The relative quantification of NS3 normalized by actin was analyzed by ImageJ software.
(TIF)

S10 Fig Interaction between ZAP and JEV 5′-UTR RNA. Different amounts (0, 1, 5, and 10 pmole) of biotin-labeled JEV 5′-UTR_197_ and 3′-UTR RNA probes were incubated with 100 μg of ZAP-S or ZAP-S-del4ZF overexpressing A549 cell extracts. The biotinylated RNA was pulled down by using streptavidin beads, and the pull-down ZAP-S-V5 (WT and del4ZFs) were then assayed by western blot.
(TIF)

S1 Table Primer sets for RT-PCR. (XLSX)

S2 Table Primer sets for amplification of DNA templates for in vitro transcription. (XLSX)
