# Supplementary figures and images for: Inhibition of Japanese encephalitis virus infection by the host zinc-finger antiviral protein
Source: PLoS Pathog. 2018 Jul 17;14(7):e1007166. doi: 10.1371/journal.ppat.1007166 (PMC6049953; doi:10.1371/journal.ppat.1007166)

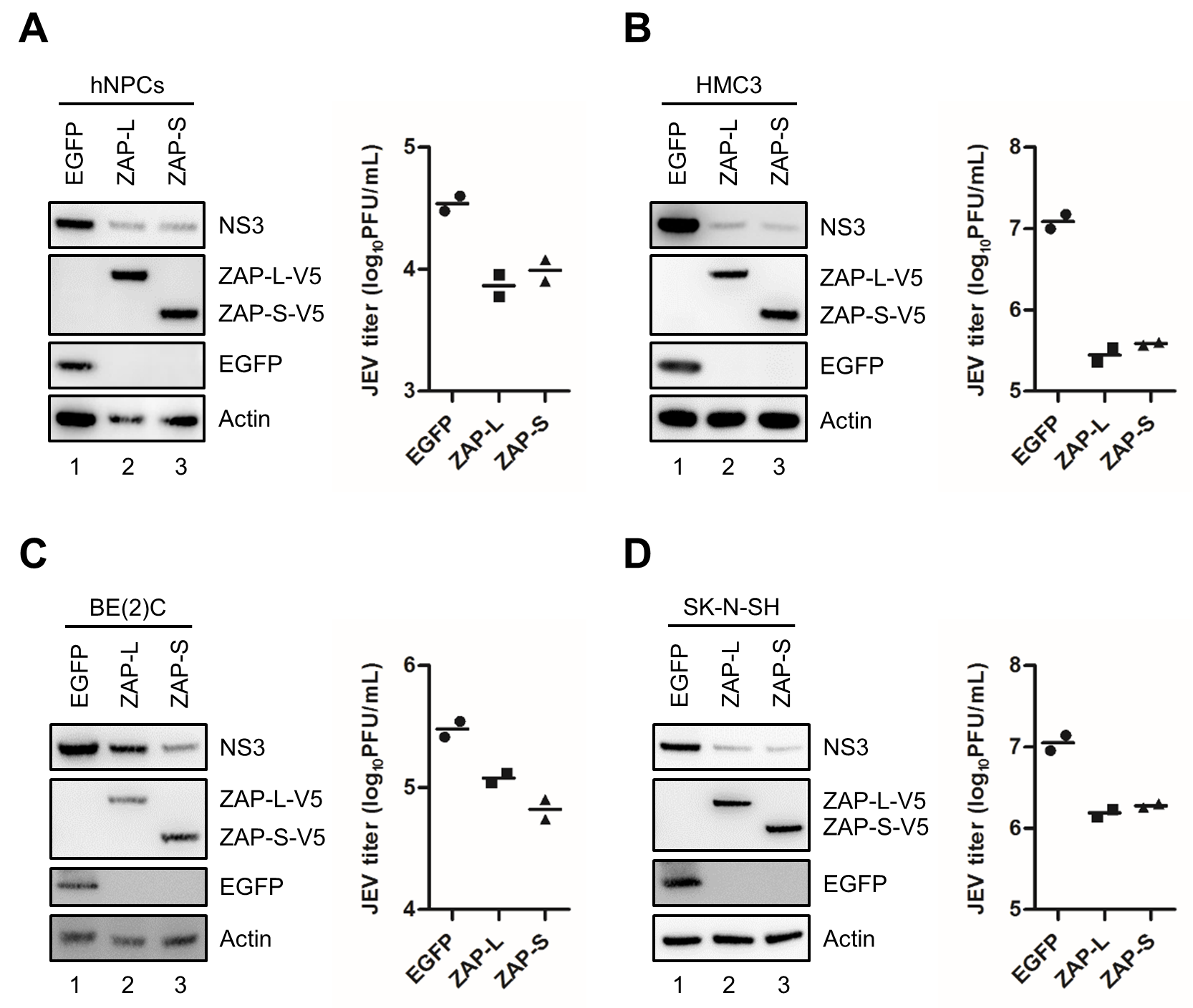

Supplement: S1 Fig — Human neuronal hNPCs (A), HMC3 cells (B), BE(2)C cells (C), and SK-N-SH cells (D) transduced with lentiviruses expressing EGFP, ZAP-L-V5, and ZAP-S-V5 were infected by JEV (MOI = 1 for hNPCs and MOI = 5 for the others) for 16 h. Cell lysates and culture supernatants were harvested to determine protein level (left panel) and viral titer (right panel) by western blot and plaque assay, respectively. Viral titration data are mean with individual data from 2 independent experiments. (TIF) [file ppat.1007166.s001.tif]

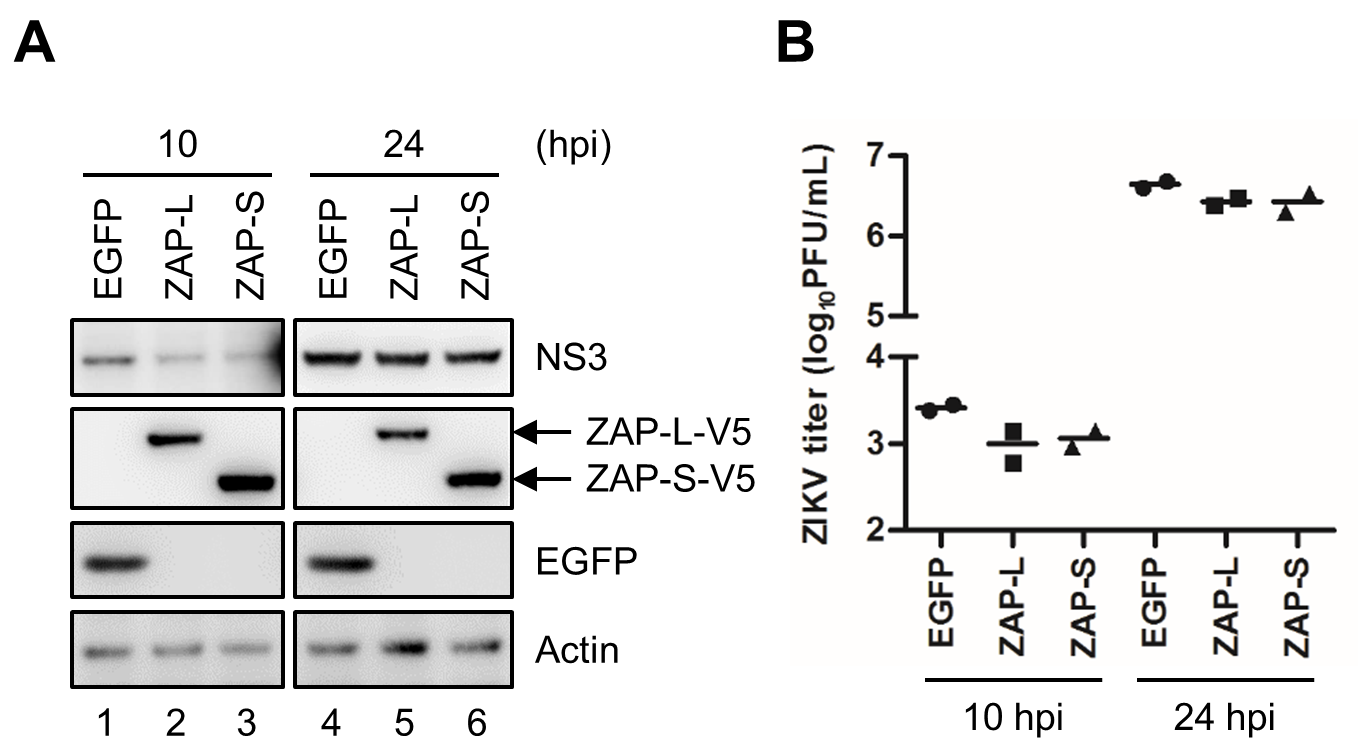

Supplement: S2 Fig — A549 cells expressing EGFP, ZAP-L-V5, and ZAP-S-V5 by lentiviral transduction were infected with ZIKV (MOI = 5) for 10 and 24 h. Collected cell lysates and culture supernatants were used to perform western blot (A) and plaque assay (B). Viral titers are mean with individual data from 2 independent experiments. (TIF) [file ppat.1007166.s002.tif]

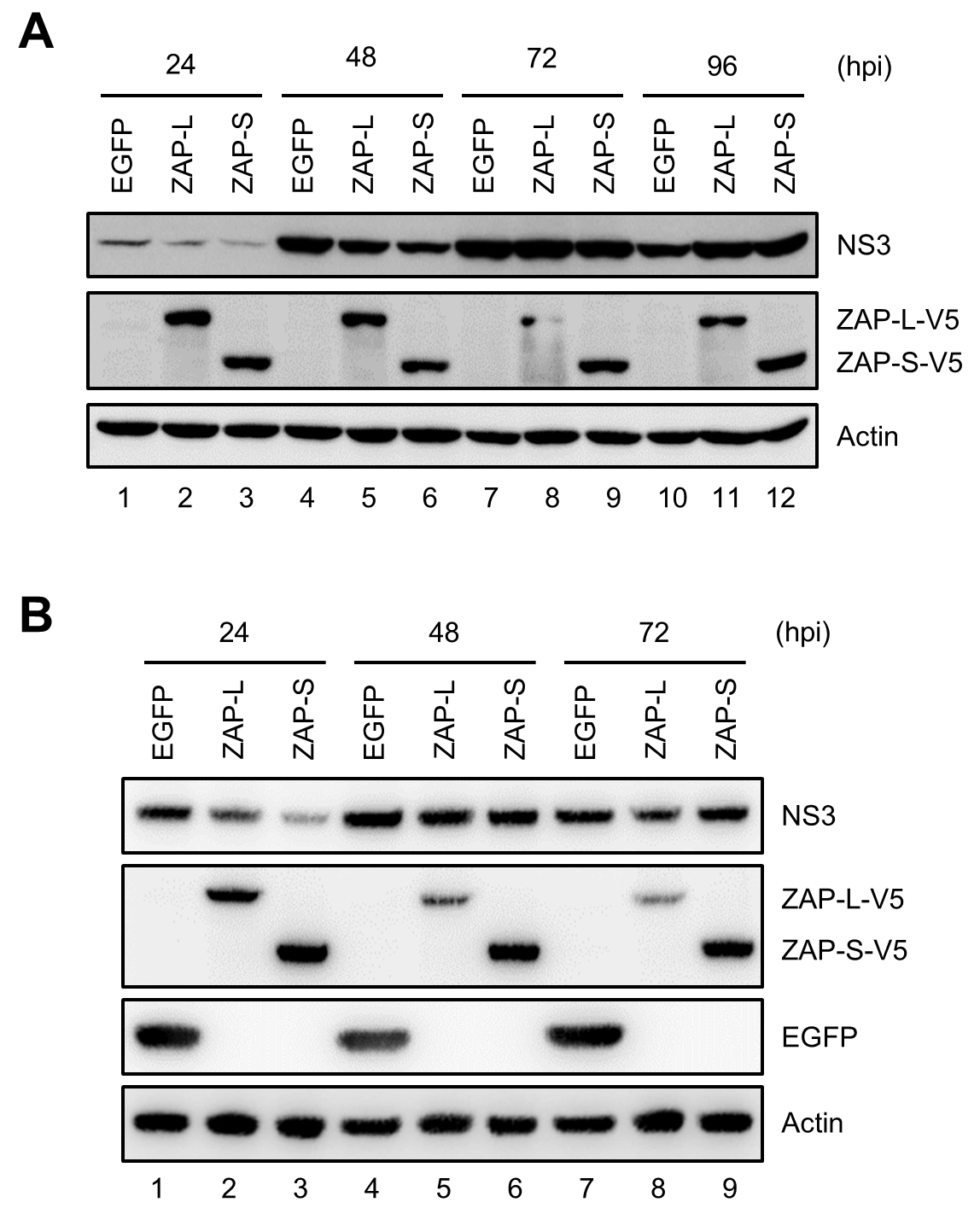

Supplement: S3 Fig — A549-EGFP, ZAP-L, and ZAP-S cells were infected with DENV (A) and ZIKV (B) (MOI = 0.1) for the indicated hours. Cell lysates were assayed for the indicated proteins by western blot. (TIF) [file ppat.1007166.s003.tif]

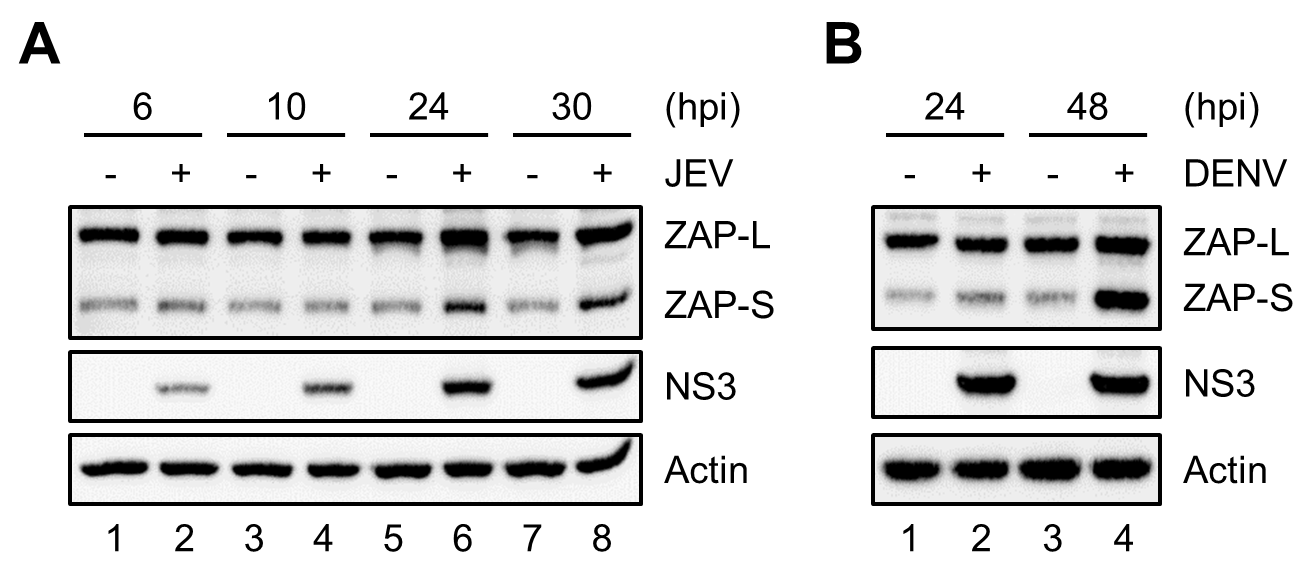

Supplement: S4 Fig — Western blot analysis for the indicated proteins in mock and JEV (A) or DENV (B) (MOI = 10) infected A549 cells at the indicated times post-infection. (TIF) [file ppat.1007166.s004.tif]

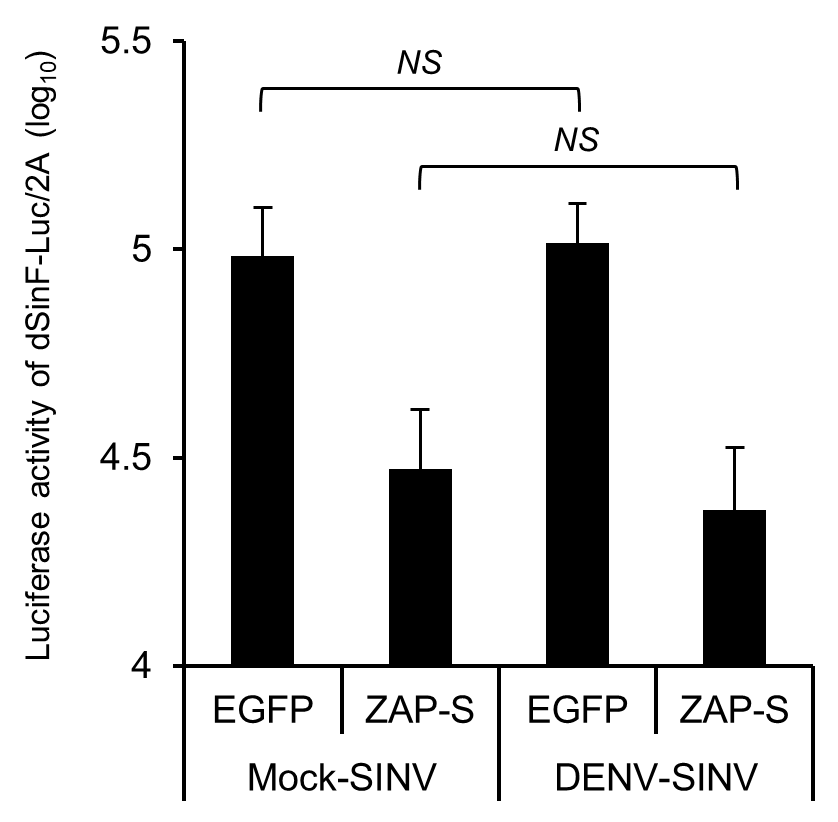

Supplement: S5 Fig — A549-EGFP and -ZAP-S cells were mock-infected or infected with DENV (MOI = 5) for 2 h, followed by infection of Sindbis virus expressing firefly luciferase (dSinF-Luc/2A) (MOI = 5) for additional 24 h. The infection of dSinF-Luc/2A was assessed by firefly luciferase assay. Data are mean ± SD (n = 3) and analyzed by two-tailed Student’s t test. NS, not significant. (TIF) [file ppat.1007166.s005.tif]

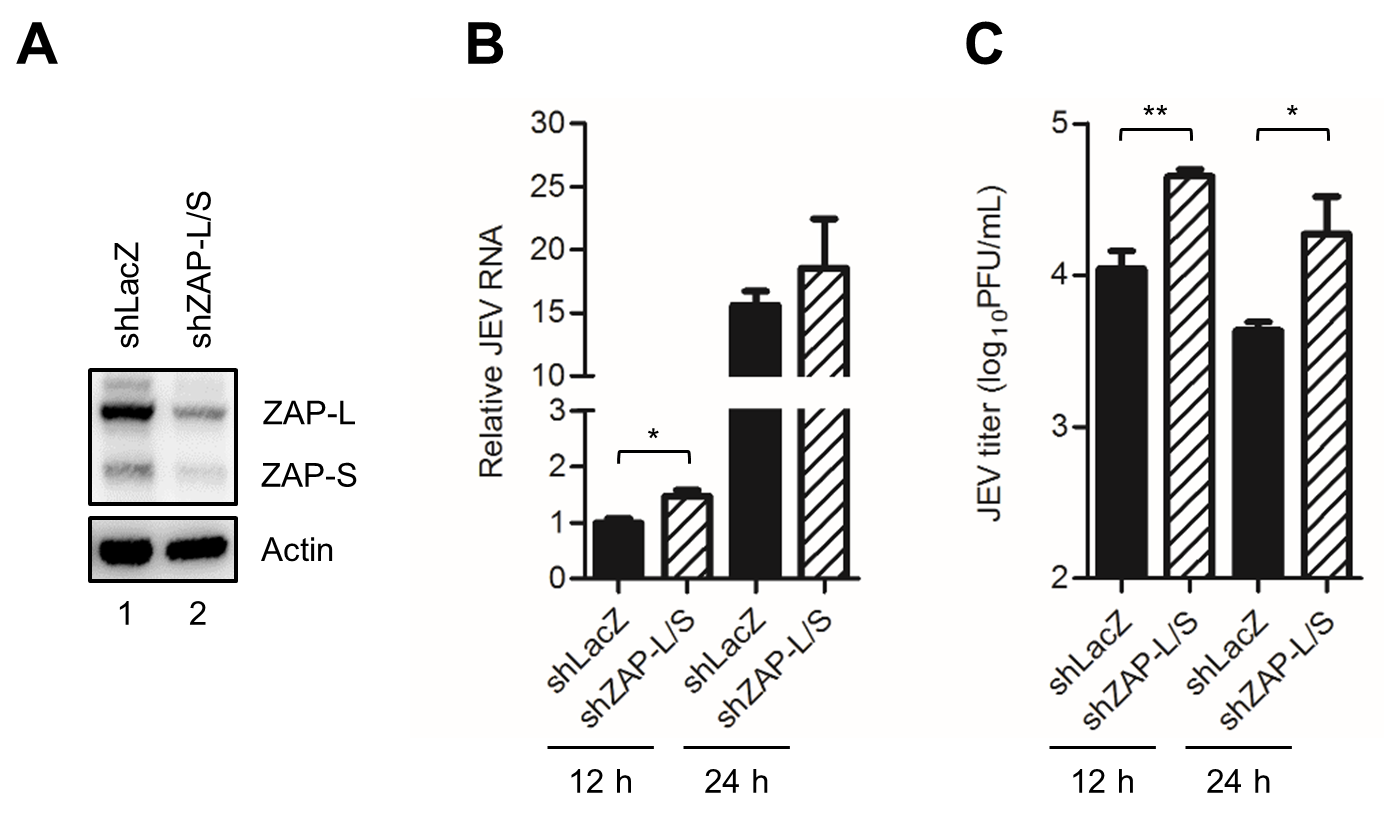

Supplement: S6 Fig — (A) Western blot analysis of ZAP isoforms and actin in BE(2)C cells with shLacZ or shZAP-L/S knockdown. (B and C) Viral RNA (B) and viral titer (C) analysis of JEV (MOI = 5) infected BE(2)C cells with shLacZ or shZAP-L/S after 12 and 24 h of infection. Relative JEV RNA level normalized by GAPDH was determined by using RT-qPCR. Viral titer was determined by plaque assay. Data are mean ± SD (n = 3) and analyzed by two-tailed Student’s t test. * P≤0.05; ** P≤0.01. (TIF) [file ppat.1007166.s006.tif]

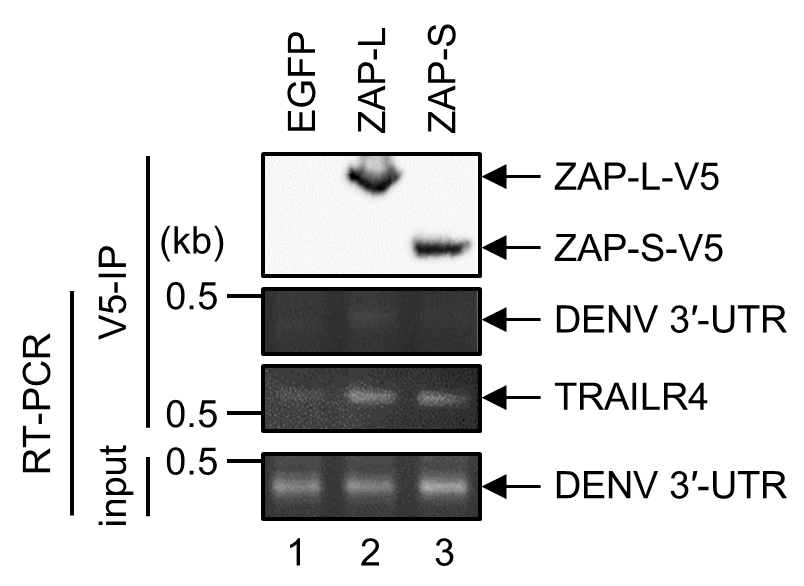

Supplement: S7 Fig — 293T/17 cells transfected with plasmids expressing EGFP, ZAP-L-V5, and ZAP-S-V5 for 24 h were infected with DENV (MOI = 10) for additional 6 h. Cell lysates were subjected to immunoprecipitation by anti-V5 agarose affinity gel, and the pull-down ZAP proteins were detected by western blot with anti-V5 antibody (upper panel). DENV RNA and cellular TRAILR4 mRNA were amplified by RT-PCR using DENV 3′-UTR and TRAILR4 specific primers (the second and third panel). RT-PCR of input viral RNA in DENV-infected cells by detection of 3′-UTR (lower panel). (TIF) [file ppat.1007166.s007.tif]

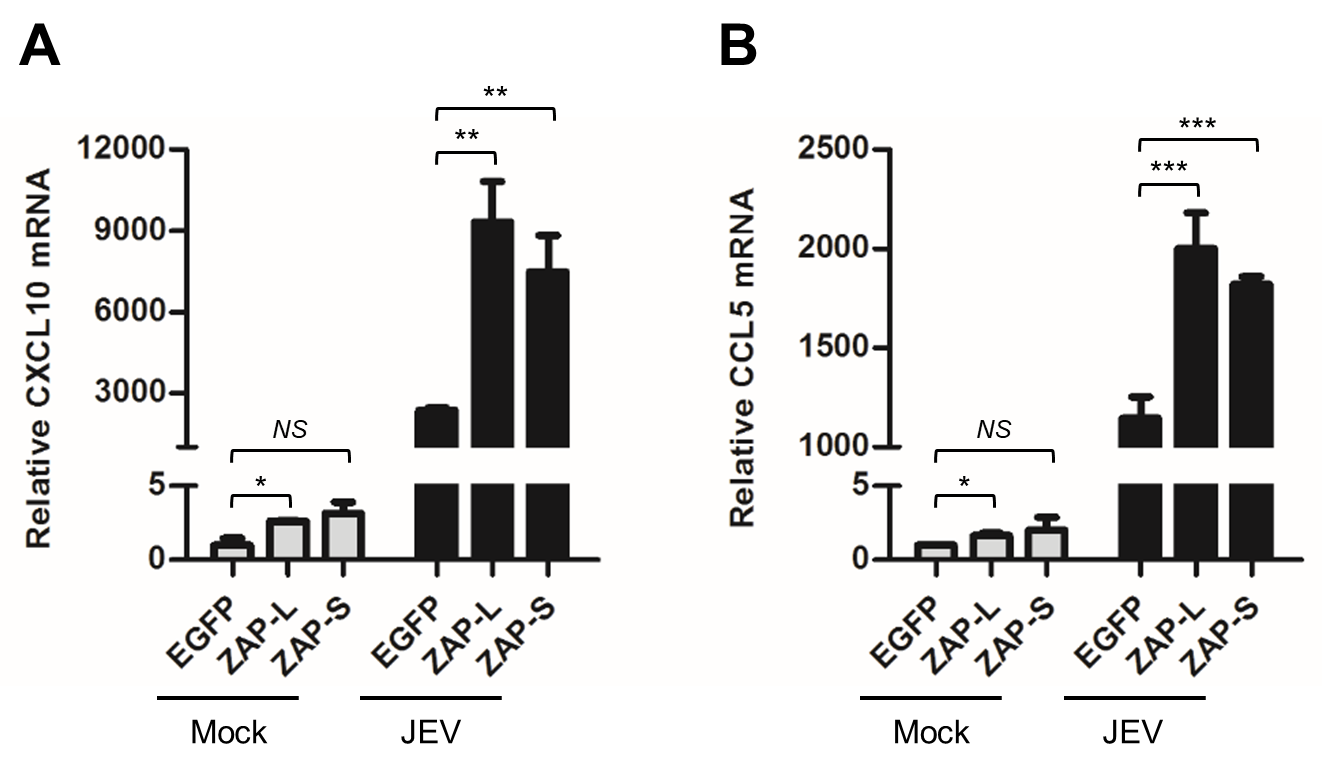

Supplement: S8 Fig — Total RNA were harvested from uninfected (Mock) and JEV (MOI = 5) infected A549-EGFP, -ZAP-L, and -ZAP-S cells at 16 hpi. The relative RNA levels of CXCL10 (A) and CCL5 (B) normalized by GAPDH were analyzed by RT-qPCR. Data are mean ± SD (n = 3) and analyzed by two-tailed Student’s t test. * P≤0.05; ** P≤0.01; *** P≤0.001; NS, not significant. (TIF) [file ppat.1007166.s008.tif]

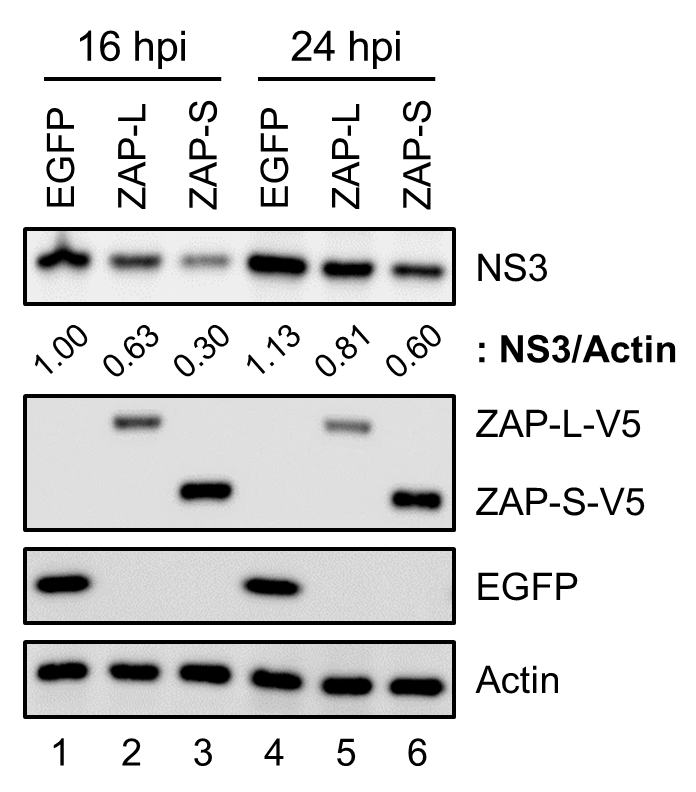

Supplement: S9 Fig — Vero-EGFP, -ZAP-L, and -ZAP-S cells established by lentiviral transduction were infected with JEV (MOI = 5) for 16 and 24 h. Western blot analysis for the indicated proteins. The relative quantification of NS3 normalized by actin was analyzed by ImageJ software. (TIF) [file ppat.1007166.s009.tif]

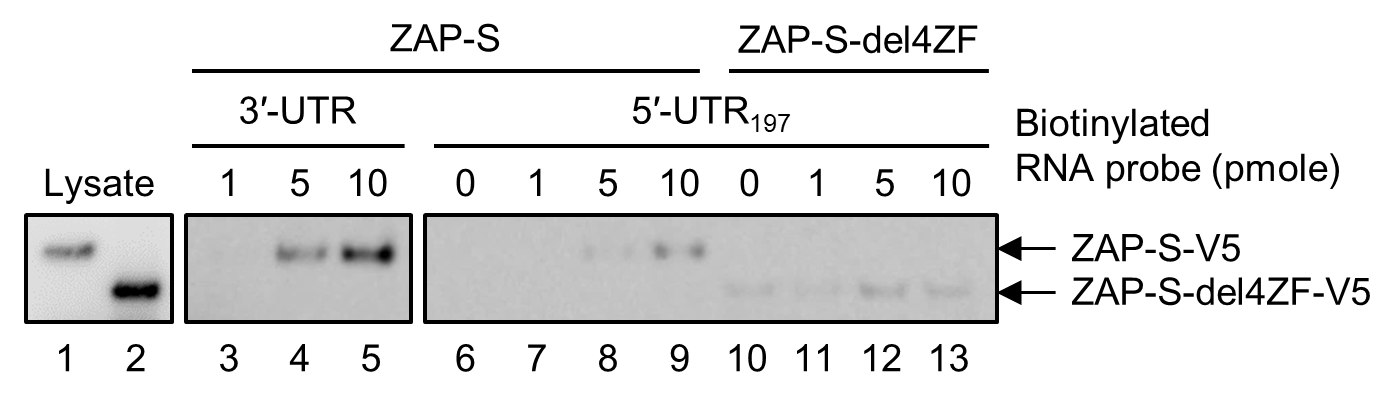

Supplement: S10 Fig — Different amounts (0, 1, 5, and 10 pmole) of biotin-labeled JEV 5′-UTR197 and 3′-UTR RNA probes were incubated with 100 μg of ZAP-S or ZAP-S-del4ZF overexpressing A549 cell extracts. The biotinylated RNA was pulled down by using streptavidin beads, and the pull-down ZAP-S-V5 (WT and del4ZFs) were then assayed by western blot. (TIF) [file ppat.1007166.s010.tif]
